# OsARM1, an R2R3 MYB Transcription Factor, Is Involved in Regulation of the Response to Arsenic Stress in Rice

**DOI:** 10.3389/fpls.2017.01868

**Published:** 2017-10-30

**Authors:** Feng-Zhu Wang, Mo-Xian Chen, Lu-Jun Yu, Li-Juan Xie, Li-Bing Yuan, Hua Qi, Ming Xiao, Wuxiu Guo, Zhe Chen, Keke Yi, Jianhua Zhang, Rongliang Qiu, Wensheng Shu, Shi Xiao, Qin-Fang Chen

**Affiliations:** ^1^State Key Laboratory of Biocontrol, Guangdong Key Laboratory of Plant Resources, Collaborative Innovation Center of Genetics and Development, School of Life Sciences, Sun Yat-sen University, Guangzhou, China; ^2^Shenzhen Research Institute, The Chinese University of Hong Kong, Shenzhen, China; ^3^School of Environmental Science and Engineering, Sun Yat-sen University, Guangzhou, China; ^4^Key Laboratory of Plant Nutrition and Fertilizer, Ministry of Agriculture, Institute of Agricultural Resources and Regional Planning, Chinese Academy of Agricultural Sciences, Beijing, China; ^5^Department of Biology, Hong Kong Baptist University, Kowloon, Hong Kong; ^6^State Key Laboratory of Agrobiotechnology, The Chinese University of Hong Kong, Shatin, Hong Kong

**Keywords:** arsenic, As transport, As uptake, MYB transcription factor, OsARM1, *Oryza sativa*

## Abstract

Bioaccumulation of arsenic (As) in rice (*Oryza sativa*) increases human exposure to this toxic, carcinogenic element. Recent studies identified several As transporters, but the regulation of these transporters remains unclear. Here, we show that the rice R2R3 MYB transcription factor OsARM1 (ARSENITE-RESPONSIVE MYB1) regulates As-associated transporters genes. Treatment with As(III) induced *OsARM1* transcript accumulation and an OsARM1-GFP fusion localized to the nucleus. Histochemical analysis of *OsARM1pro::GUS* lines indicated that *OsARM1* was expressed in the phloem of vascular bundles in basal and upper nodes. Knockout of *OsARM1* (*OsARM1-KO* CRISPR/Cas9-generated mutants) improved tolerance to As(III) and overexpression of *OsARM1* (*OsARM1-OE* lines) increased sensitivity to As(III). Measurement of As in As(III)-treated plants showed that under low As(III) conditions (2 μM), more As was transported from the roots to the shoots in *OsARM1-KOs*. By contrast, more As accumulated in the roots in *OsARM1-OEs* in response to high As(III) exposure (25 μM). In particular, the As(III) levels in node I were significantly higher in *OsARM1-KOs*, but significantly lower in *OsARM1-OEs*, compared to wild-type plants, implying that OsARM1 is important for the regulation of root-to-shoot translocation of As. Moreover, *OsLsi1, OsLsi2*, and *OsLsi6*, which encode key As transporters, were significantly downregulated in *OsARM1-OEs* and upregulated in *OsARM1-KOs* compared to wild type. Chromatin immunoprecipitation-quantitative PCR of *OsARM1-OEs* indicated that OsARM1 binds to the conserved MYB-binding sites in the promoters or genomic regions of *OsLsi1, OsLsi2*, and *OsLsi6* in rice. Our findings suggest that the OsARM1 transcription factor has essential functions in regulating As uptake and root-to-shoot translocation in rice.

## Introduction

Arsenic (As) occurs in many minerals, usually in combination with sulfur and metals, and can be found in two inorganic forms, arsenite [As(III)] and arsenate [As(V)]. As(III) causes detrimental effects on cells by binding to sulfhydryl groups in proteins and blocking their activity, whereas As(V) functions as a phosphate analog, affecting several essential biological processes, including ATP synthesis and phosphorylation (Li et al., 2016). As is a group I carcinogen and a highly toxic, chronic poison in humans, causing skin lesions, keratosis, hyperpigmentation, diabetes, and other conditions (Argos et al., 2010). The majority of As-related diseases arise from contamination of underground water used for drinking and irrigating crops (Smith et al., 2002). This problem is especially serious in developing countries in South America and Southeast Asia (Brammer and Ravenscroft, 2009; Nicolli et al., 2012; Mirlean et al., 2014). Increasing evidence also indicates that As has chronic effects and accumulates at the top of the food chain (Li et al., 2011).

High levels of As in agricultural soils may increase human As exposure through the consumption of contaminated crops or vegetables (Zhao et al., 2010). Accumulation of As in soil usually results from irrigation with As-containing underground water, the application of herbicides or insecticides that contain As, or regional mining activities (Verbruggen et al., 2009). Therefore, reducing As accumulation in underground water and soil used for crop production is essential to protect humans from As poisoning.

Exposure of plants to excess As can cause toxicity in the plants, either directly or indirectly, including symptoms such as production of reactive oxygen species (ROS) and lipid peroxidation (Stoeva et al., 2003; Sytar et al., 2013). In plants, As can inhibit seed germination (Li et al., 2007), reduce shoot height (Abedin et al., 2002), suppress root elongation (Shri et al., 2009), and reduce photosynthesis and grain yields (Rahman et al., 2007). Plants have evolved several adaptive mechanisms to cope with As stress, such as forming complexes between As and thiol-rich peptides such as glutathione and phytochelatins (Meharg and Hartley-Whitaker, 2002), sequestering As into the vacuole, and translocating As from the root to the shoot (Zhao et al., 2009).

Rice, a major crop in many areas with severe As contamination, can efficiently assimilate As from paddy soils; therefore, rice likely represents a primary dietary source of inorganic As (Meharg et al., 2009; Li et al., 2011). In flooded paddy soils, As(III), the predominant As species, can be taken up into rice roots by the nodulin 26-like intrinsic (NIP) aquaporin OsNIP2;1 (Lsi1) and effluxed toward the stele for xylem loading by the silicon efflux transporter Lsi2 (Ma et al., 2006, 2007). Lsi1 and Lsi2 localize to the plasma membrane in the exodermis and endodermis. Lsi1 localizes on the distal side of root cells and Lsi2 localizes on the proximal side (Ma et al., 2008). In addition, rice Lsi1 mediates the uptake of methylated As species such as mono-methylarsonic acid and dimethylarsinic acid (Li et al., 2009). Excess As not eliminated by plasma membrane transporters is either stored in root vacuoles or translocated to the shoots and delivered to other organs such as nodes and grains (Zhao et al., 2010; Li et al., 2016). Node phloem cells may accumulate As and participate in As transport, suggesting their potential role in distributing As to rice grains (Moore et al., 2014). The *Lsi2* transcript accumulates in roots and nodes; roots represent hubs for the storage and distribution of As and other mineral nutrients in graminaceous plants (Yamaji and Ma, 2014). Recent findings indicate that upon exposure of excised panicles of the *lsi2* mutant to As(III), more As is distributed to the node and flag leaf but less is distributed to the grain compared to wild type, indicating that Lsi2 plays an important role in regulating As(III) distribution in rice nodes (Chen et al., 2015).

Lsi6 (OsNIP2;2) functions as a silicon (Si) transporter in rice and is found in xylem parenchyma cells of the leaf sheath, leaf blade, and xylem transfer cells in node I (Yamaji et al., 2008; Yamaji and Ma, 2009). Moreover, Lsi6 shows polar localization at the side facing toward the vessel, where it transports Si out of the xylem and subsequently affects Si distribution in rice shoots (Yamaji et al., 2008). Additionally, knockout of *Lsi6* increased Si accumulation in the flag leaf and thus decreased Si accumulation in the panicles (Yamaji and Ma, 2009). Even though Lsi6 does not contribute substantially to As uptake by rice roots, it had As(III) transport activity when expressed in oocytes (Ma et al., 2008) and its expression was reduced in response to As(III) treatment (Yu et al., 2012).

Many studies have explored As uptake, cellular partitioning, and long-distance translocation (Li et al., 2016). For example, the tonoplast-localized ATP-binding cassette (ABC) transporter OsABCC1 controls arsenic transport into rice grains by sequestering the As(III)-PC complex in the vacuoles of the phloem companion cells at the nodes (Song et al., 2014). Similarly, two *Arabidopsis* ABC transporters (AtABCC1 and AtABCC2) transport As into the vacuole, revealing their essential roles in As detoxification (Song et al., 2010).

Despite emerging knowledge on As transport, the regulatory mechanisms underlying the plant response to As stress remain unclear. Recently, an arsenate-responsive transcription factor (WRKY6) was found to regulate the expression of arsenate/phosphate transporters in *Arabidopsis* (Castrillo et al., 2013). Our previous global transcriptome analysis in rice treated with As(III) identified a number of As(III)-responsive genes involved in various biological processes, including heavy metal transport, jasmonate signaling, and transcriptional regulation (Yu et al., 2012). Among these, the expression of a novel R2R3 MYB transcription factor gene, *OsARM1* (A*RSENITE-*R*ESPONSIVE* M*YB*1; LOC_Os05g37060), is strongly induced by As(III) treatment, suggesting its potential role in the transcriptional regulation of As responses. MYB proteins constitute a diverse group of transcription factors in plants and have a conserved DNA-binding domain (Jin and Martin, 1999). The functions of MYB transcription factors in plants have been extensively investigated (Jin and Martin, 1999; Chen et al., 2006). These transcription factors are associated with plant responses to various biotic and abiotic stresses, such as phosphate starvation, UV-B irradiation, chilling and freezing temperatures, and salt and drought stress (Jin et al., 2000; Rubio et al., 2001; Agarwal et al., 2006; Dai et al., 2007; Shin et al., 2011).

In the current study, we found that the expression of *OsARM1* was significantly induced in response to As(III) stress in rice. Histochemical GUS staining assays of plants carrying a promoter::GUS fusion construct indicated that *OsARM1* was predominately expressed in the basal and upper nodes of rice plants, with intense staining in the phloem region. Genetic, phenotypic, and biochemical analyses revealed that OsARM1 was involved in the regulation of As tolerance in rice, possibly by modulating the uptake and root-to-shoot translocation of As *in planta*.

## Materials and methods

### Plant materials, growth conditions, and arsenic treatment

The *Oryza sativa* cultivars Nipponbare (NPB), Dongjing (DJ), and SSBM were used in this study. The rice T-DNA insertion seed pool used to isolate the *osarm1* mutant (PFG_3A-12233.R) was obtained from Rice T-DNA Insertion Sequence Database (developed by Dr. Gynheung An, Department of Plant Systems Biotech, Kyung Hee University, Republic of Korea) (Jeon et al., 2000; Jeong et al., 2006). The T-DNA insertion site in the *osarm1* mutant was identified using *OsARM1* gene-specific primers XS830/XS831 paired with the T-DNA right border primer PGVRB. The primers used in this article are listed in Table S1. Generation of the *OsARM1-KO* mutants by CRISPR/Cas9 editing is described below.

All rice seeds were surface sterilized with 75% ethanol for 40 s, followed by 20% NaClO for 20 min. After being rinsed ~five times with sterile distilled water, the seeds were germinated on half-strength Murashige and Skoog (1/2 MS) medium at 25°C under a 12-h light/12-h dark photoperiod.

Two-week-old rice seedlings were transferred to Kimura B nutrient solution with or without 2, 5, 25, or 40 μM As(III) for arsenite tolerance analysis. After 7 or 14 days of treatment, the phenotypes were recorded by measuring the root lengths and heights of the seedlings.

For As(III) treatment of *Arabidopsis*, seeds of wild-type (Col-0) and transgenic plants expressing OsARM1-GFP were germinated on 1/2 MS medium for 1 week under normal growth conditions. The seedlings were subsequently transferred to 1/2 MS medium or 1/2 MS medium containing 10 or 20 μM As(III) for further growth. Photographs were taken and dry weights were recorded at 2 weeks after treatment.

### RNA extraction and gene expression analysis

The expression level of *OsARM1* was tested in total RNA extracted from various organs (roots, stems, leaf blades, panicles, and grains) of 14-week-old soil-grown plants with the TRIzol RNA extraction kit (Invitrogen, Thermo Fisher Scientific) following the manufacturer's instructions and RT-PCR using the primers XS147 and XS148 with *OsACTIN1* (XS1043 and XS1044) as a reference gene.

To test the expression of *OsARM1* and transporter genes in response to As, total RNA was extracted from various tissues of 2-week-old seedlings. The isolated RNA was reverse transcribed to obtain first-strand cDNA using PrimeScript RT reagent with gDNA eraser kit (Takara). The qRT-PCR was performed using gene-specific primers (Table S1) and SYBR Premix Ex Taq II (Takara) on a StepOne Plus real-time PCR system (Applied Biosystems). *OsACTIN1* (Wang et al., 2012) or *OsGAPDH* (Pabuayon et al., 2016) was used as an internal reference.

### Generation of *OsARM1pro::GUS* transgenic rice and histochemical analysis

To generate the *OsARM1pro::GUS* plasmid, about 1.5 kb of sequence in the promoter region of *OsARM1*, including 19 bp downstream of the start codon (ATG) of *OsARM1* was amplified from rice genomic DNA by high-fidelity PCR using primer pairs XS489 and XS490 (Table S1). This fragment contained 1.5 kb of the promoter region of *OsARM1*, 19 bp from the first exon of *OsARM1*, and 8 bp from the vector, which was translationally in-frame with the GUS fusion. This PCR fragment was then cloned into the *Xcm*I site of the pCXGUS-P vector (Chen et al., 2009). The insert was validated by sequencing and the confirmed plasmid was introduced into *Agrobacterium tumefaciens* strain LBA4404 and transformed into the wild-type rice cultivar SSBM using a rapid and efficient Agrobacterium-mediated transformation method (Toki, 1997). Putative transformants selected on 1/2 MS medium containing hygromycin (50 mg·L^−1^) were verified by PCR using a promoter-specific forward primer and the GUS-specific reverse primer GUS-SEQ (Table S1). Subsequently, the T_3_ homozygous lines were sown on 1/2 MS medium containing hygromycin and the resistant plants were used for further analysis.

Homozygous transgenic lines expressing *OsARM1*_*pro*_*::GUS* were stained to detect GUS activity as described previously (Zheng et al., 2012; Chen et al., 2013). The samples were immersed in GUS staining solution [50 mM K_4_Fe(CN)_6_·3H_2_O, 50 mM K_3_[Fe(CN)_6_], 0.2 M NaH_2_PO_4_·2 H_2_O, 0.2 M Na_2_HPO_4_·12 H_2_O, 10% Triton X-100, 100 mg·mL^−1^ X-Gluc] and vacuum infiltrated for 20 min, followed by 8 h to overnight incubation at 37°C, depending on the desired staining intensity. After staining, the tissues were rinsed several times with 75% ethanol until the chlorophyll was removed. GUS-stained seedling tissues were observed under a fluorescence stereo-microscope (SteREO Lumar. V12). The tissues were then cleared with HCG solution (chloral hydrate/water/glycerol 8:3:1) and observed and photographed under a fluorescence microscope (Leica DM5000B).

### Subcellular localization of OsARM1-GFP protein

The full-length *OsARM1* cDNA was amplified using primer pairs XS260 and XS261 (Table S1) and fused to the N terminus of green fluorescent protein (GFP) in the pBI-eGFP vector (Xiao et al., 2008). For the transient expression analyses, the empty vector (which expresses GFP) and the OsARM1-GFP construct were transiently expressed in rice protoplasts isolated from 8-day-old green seedlings as described previously (Zhang et al., 2011). The AtARF4-RFP plasmid (Piya et al., 2014) was used as a nuclear marker for co-expression with GFP or OsARM1-GFP. To generate stable transgenic lines, the OsARM1-GFP construct was introduced into *A. tumefaciens* LBA4404 and transformed into wild-type *Arabidopsis* Col-0 by the floral dip method (Clough and Bent, 1998). To further confirm the localization of OsARM1-GFP in the nuclei, the leaves of 1-week-old transgenic *Arabidopsis* expressing OsARM1-GFP and the GFP control were infiltrated with phosphate-buffered saline (PBS, pH 7.4) containing 2 ng·μL^−1^ 4′, 6′-diamidino-2-phenylindole (DAPI) for 10 min and washed several times with PBS (Wang et al., 2016). The fluorescence was detected by confocal laser scanning microscopy (Zeiss 7 DUO NLO).

### Generation of *OsARM1-KO* and *OsARM1-OE* transgenic lines

To generate *OsARM1-KO* mutants by CRISPR/Cas9, 19, or 20 bp of conserved sequence from the *OsARM1* regions encoding the MYB-type HTH DNA-binding domains were selected as targets (Table S1). The sequences were ligated to the pYLCRISPR/gRNA vector, followed by ligation to pYLCRISPR/Cas9-MTmono vector after dual-nested PCR as previously described (Ma et al., 2015). The plasmids were introduced into *A. tumefaciens* strain LBA4404 and transformed into wild-type rice NPB using the traditional rice transformation method (Toki, 1997). The genome editing of *OsARM1-KO* transgenic rice lines was confirmed by sequencing PCR products amplified with primer pairs M4T1-F/M4T1-R and M4T2-F/M4T2-R (Table S1).

To generate *OsARM1* overexpression (*OsARM1-OE*) lines, the entire cDNA sequence of *OsARM1* was amplified using primer pairs XS491 and XS492 and cloned into the *Xcm*I site of the binary vector pCXSN-Myc (Chen et al., 2009). The plasmids (constructed as described above) were also introduced into *A. tumefaciens* strain LBA4404 and transformed into wild-type rice NPB using the traditional rice transformation method (Toki, 1997). The expression levels of *OsARM1* in the *OsARM1-OE* transgenic lines were determined by RT-PCR analyses.

### Determination of As content

Two-week-old NPB, *OsARM1-KOs*, and *OsARM1-OEs* seedlings were treated with Kimura B solution contained 2 or 25 μM As(III) for 7 days, and root and shoot samples were collected separately. To remove the adsorbed As from the root surface, the roots were washed three times with distilled water. Then the roots and shoots were collected separately and dried at 65°C for 2 days. To determine the As levels in various aboveground organs, NPB, *OsARM1-KOs*, and *OsARM1-OEs* rice plants were grown in As-containing soil (20 mg As per 1 kg soil) until the maturity. Samples were harvested from various organs as previously described (Yamaji and Ma, 2014) and dried at 65°C for 2 days. After digestion with 10 mL of 4:1 HNO_3_/HClO_4_ at 180°C for 8 h (Chen et al., 2005), the total As contents were determined using atomic fluorescence spectrometry (AFS-8220).

### ChIP assays

ChIP assays were performed as described previously (Yamaguchi et al., 2014) using 2-week-old *OsARM1-OE* transgenic rice seedlings and *Arabidopsis* seedlings expressing the OsARM1-GFP fusion protein (*GFP-9*). After coating with anti-Myc (Abiocode) or anti-GFP (Abiocode) antibodies (Cell Signaling Technology), the protein/DNA complexes were immunoprecipitated with Dynabeads Protein G (Invitrogen) for at least 4 h at 4°C. The precipitated DNA was purified using a DNA purification kit (Qiagen), and the enriched DNA fragments were subjected to qPCR using the specific primers listed in Table S1. The *OsACTIN1* and *AtACTIN2* promoters were used as negative controls. All ChIP assays were repeated three times with similar results.

## Results

### *OsARM1* is induced by As(III) treatment

**Figure 1:**
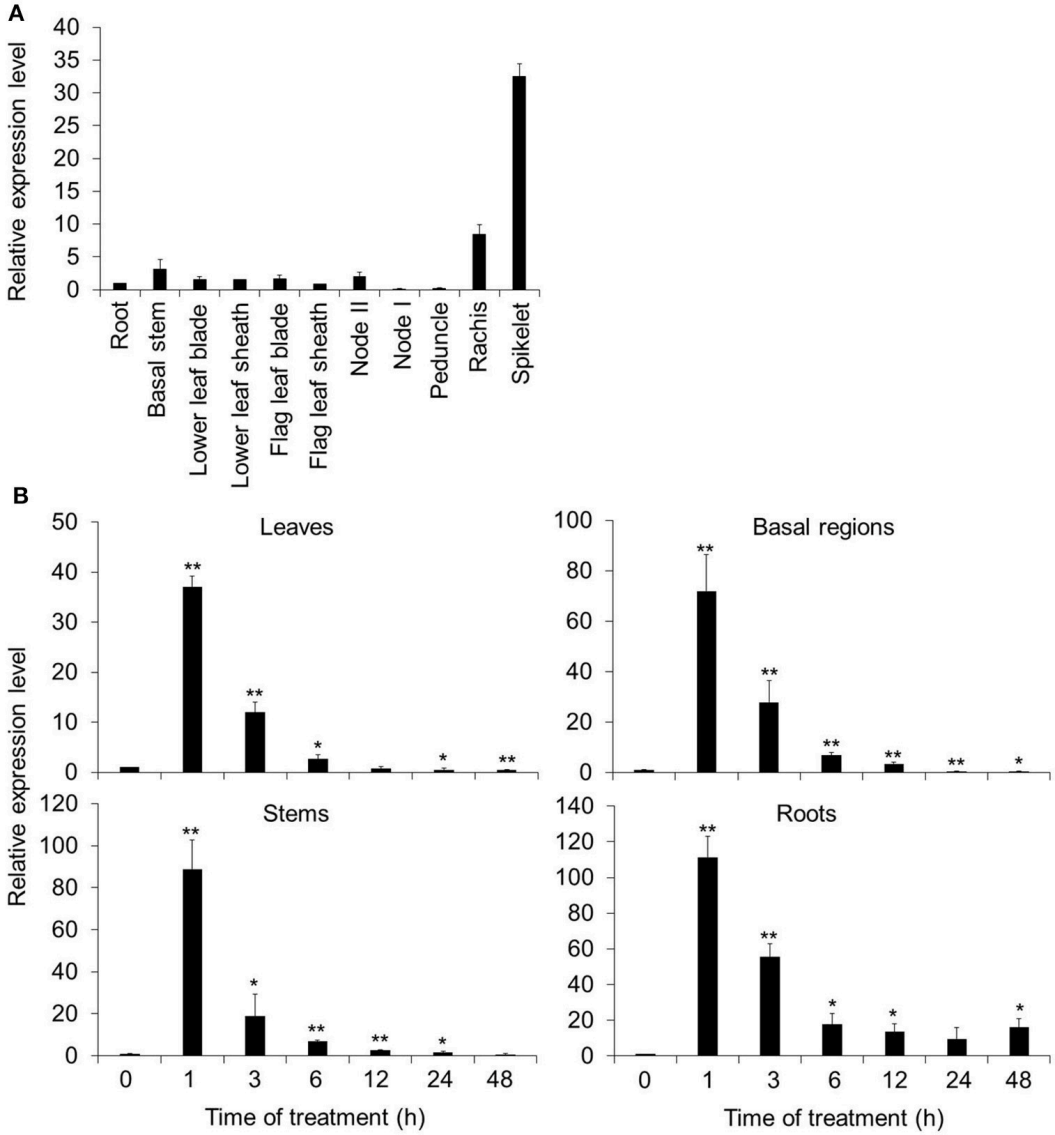
Spatial and temporal expression patterns of *OsARM1*. **(A)** Distribution of *OsARM1* transcript in different organs. Total RNA was extracted from various samples (roots, basal stems, lower leaf blades, lower leaf sheaths, flag leaf blades, flag leaf sheaths, node II, node I, peduncles, rachises, and spikelets) harvested from 14-week-old wild-type Nipponbare (NPB). **(B)** Induction of *OsARM1* expression by As(III) treatment. Total RNA was isolated from leaves, stems, basal regions, and roots of 2-week-old NPB treated with 50 μM As(III). The samples were harvested at 0, 1, 3, 6, 12, 24, and 48 h after treatment. The experiments were repeated three times (biological replicates, each replicate was pooled from 12 individual plants) with similar results, and representative data from one replicate are shown. Data are means ± SD (*n* = 3) of three technical replicates. *OsACTIN1* was used as a reference gene. Asterisks indicate significant differences from wild type (^*^P < 0.05, ^**^P < 0.01 by Student's *t*-test).

The expression pattern of *OsARM1* was first investigated by quantitative reverse-transcription PCR (qRT-PCR) analysis, in different organs of 14-week-old wild-type rice (*O. sativa* L. *japonica*. cv. Nipponbare, NPB) grown in soils. The *OsARM1* transcript was widely distributed in all organs with high levels in the rachis and spikelets (Figure 1A).

To investigate the potential role of OsARM1 in the plant response to As(III) stress, we next tested whether As could induce *OsARM1* expression. To this end, we subjected 2-week-old wild-type rice seedlings (NPB) to treatment with 50 μM As(III) and investigated the expression patterns of *OsARM1* by qRT-PCR in various tissues including leaves, stems, basal regions, and roots. *OsARM1* expression was rapidly induced after As(III) treatment at various time points, peaking at 1-h of As(III) exposure (Figure 1B). In particular, *OsARM1* transcript levels increased 37.1-, 88.8-, 71.9-, and 111.3-fold in As(III)-treated leaves, stems, basal regions, and roots, respectively, compared with the untreated controls (Figure 1B). The As(III)-inducible expression of *OsARM1* is consistent with our previous findings (Yu et al., 2012). In addition, *OsARM1* expression levels were significantly reduced after 3–48 h of As(III) treatment (Figure 1B).

**Figure 2:**
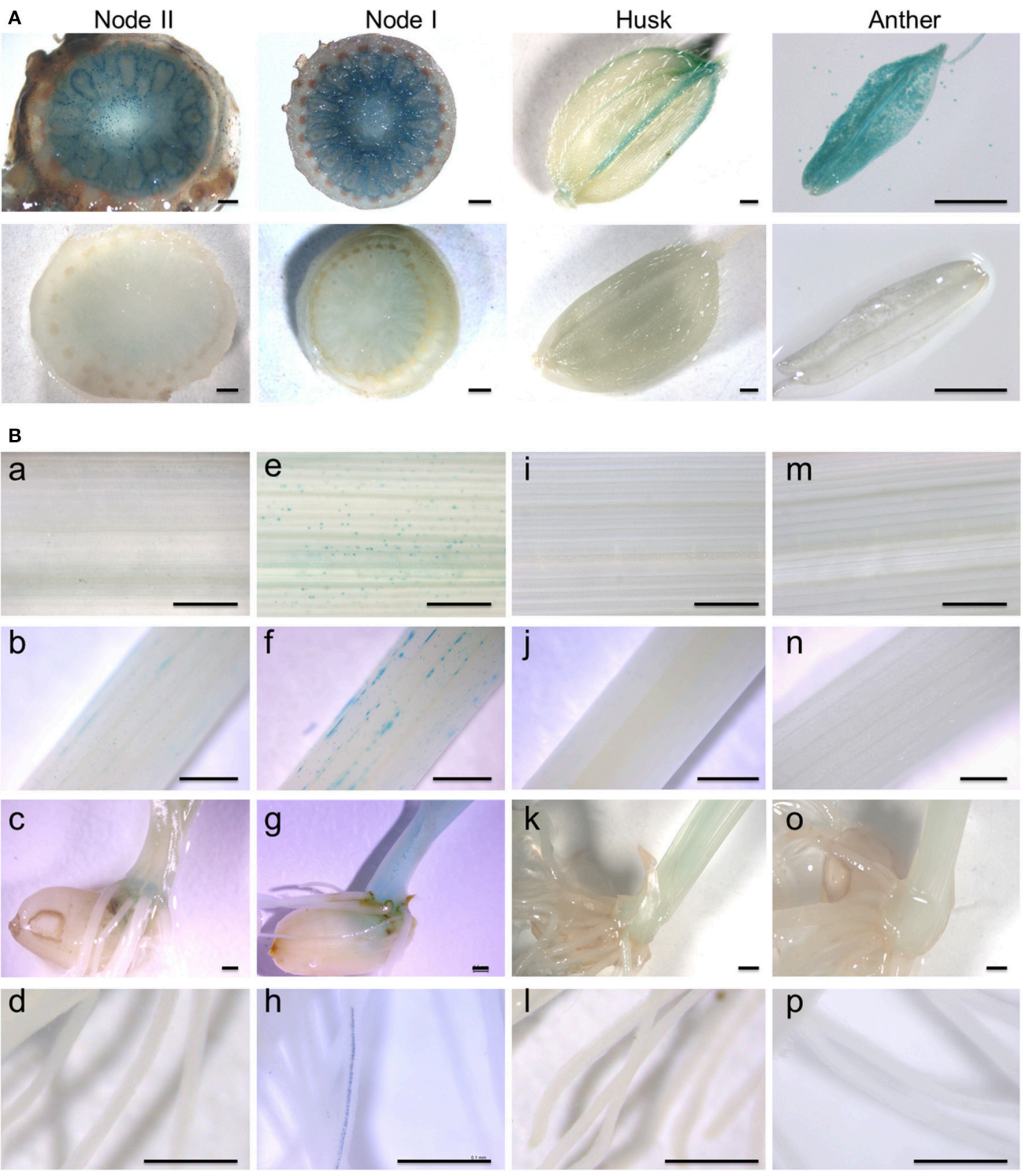
Spatial and temporal expression of the *OsARM1*_*pro*_*::GUS* construct. **(A)** Histochemical GUS staining of transgenic rice expressing *OsARM1*_*pro*_*::GUS* showing high levels of GUS signal in the enlarged vascular bundles of node I and node II, the vessel tissues of the husk, and the anther (upper images). GUS staining of wild-type SSBM was used as a negative control (bottom images). **(B)** The expression patterns of *OsARM1*_*pro*_*::GUS* upon exposure to As(III). Various organs were collected from two-week-old seedlings at the vegetative growth stage. The *OsARM1*_*pro*_*::GUS* seedlings were treated with 50 μM As(III), and the samples were harvested at 0 and 6 h after treatment. Images in (e–h) show As(III)-treated samples of leaves (e), stems (f), basal regions (g), and roots (h) of *OsARM1*_*pro*_*::GUS* seedlings. Images in (a–d) show the corresponding untreated controls. Wild-type SSBM samples harvested from As(III)-treated (m–p) or untreated (i–l) plants were used as negative controls. Bars = 500 μm.

To further elucidate the expression pattern of *OsARM1*, we generated transgenic rice plants expressing *OsARM1* promoter fusions with the β-glucuronidase (GUS) reporter (*OsARM1*_*pro*_*::GUS*). Histochemical GUS staining showed that, under normal growth conditions, *OsARM1*_*pro*_*::GUS* was expressed primarily in the enlarged vascular bundles of node I and node II, the vessel tissues of the husk, and the anther at the reproductive growth stage (Figure 2A, upper images). However, *OsARM1*_*pro*_*::GUS* expression was considerably lower in leaves, stems, basal regions, and roots at the vegetative growth stage (Figure 2B, panels a–d). Given that the expression of *OsARM1pro::GUS* is extremely low under normal growth conditions (Figure 2B, panels a–d), we investigated the induction of *OsARM1* promoter under As(III) stress (Figure 2B, panels e–h). After exposure to 50 μM As(III) for 6 h, *OsARM1*_*pro*_*::GUS* expression was activated in the above-mentioned tissues (Figure 2B, panels e–h). On the other hands, no GUS signals in the tested tissues of control samples were observed under normal conditions (Figure 2A, lower images and Figure 2B, panels i–l) or under As(III) stress conditions (Figure 2B, panels m–p).

We further investigated the cell specificity of *OsARM1* expression in roots, leaves, and stems by observing tissue slices by fluorescence microscopy. GUS activity was detected in the xylem transfer cells of roots (Figure S1A), the vessels of leaves (Figures S1B,D) and the tracheids of stems (Figure S1C). Together, our results reveal that *OsARM1* expression is induced by As(III) treatment and that *OsARM1* transcript accumulates specifically in vascular tissues.

### OsARM1-GFP primarily localizes to the nucleus

**Figure 3:**
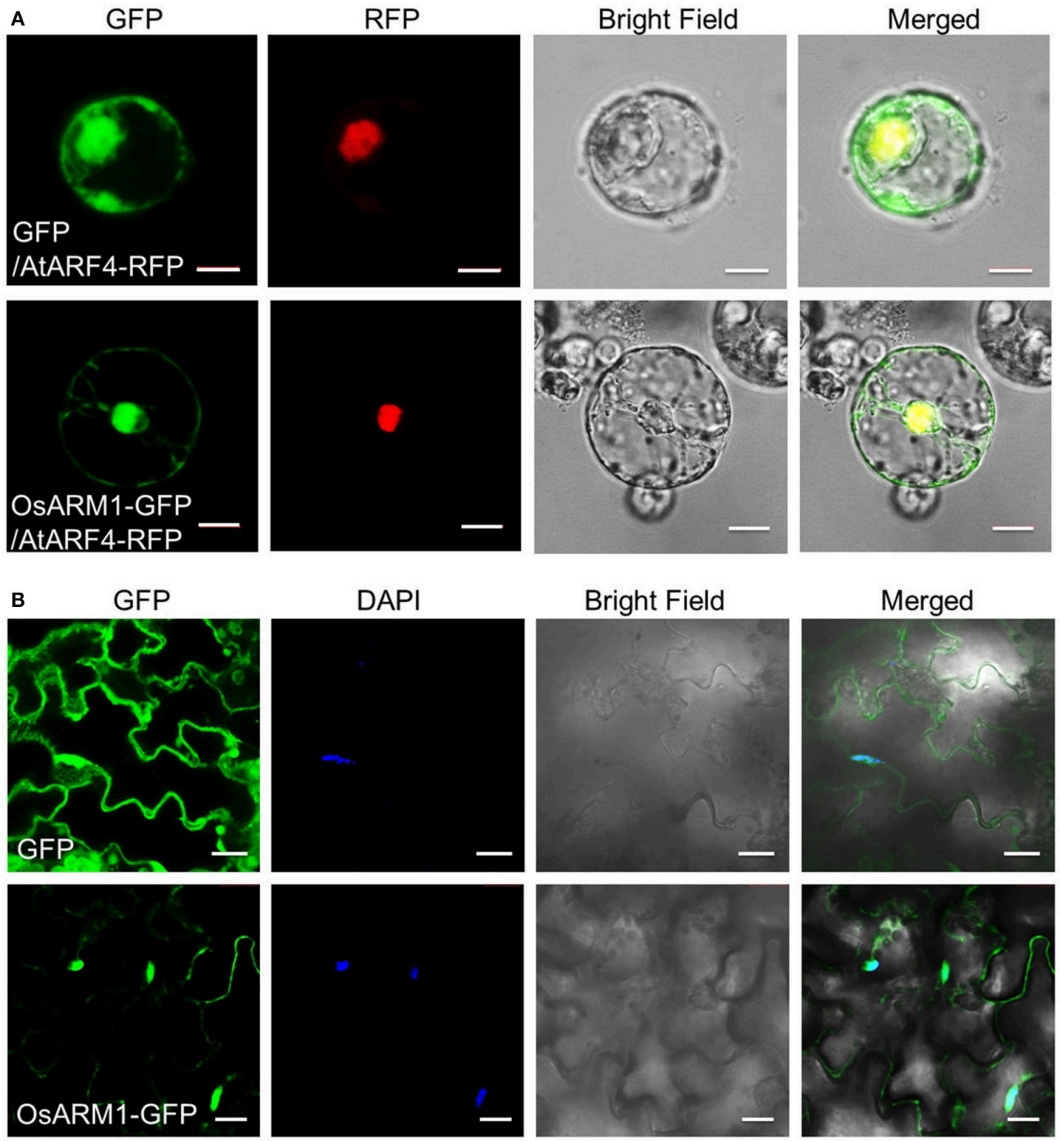
OsARM1-GFP mainly localizes to the nucleus. **(A)** 8-day-old rice green seedlings (stem and sheath) were used for protoplast isolation. The 35S::GFP empty vector pBI-eGFP and nuclear marker AtARF4-RFP or vector 35S::OsARM1-GFP and AtARF4-RFP were transiently co-expressed in protoplasts. Bars = 10 μm. **(B)** Leaf epidermal cells from 1-week-old transgenic *Arabidopsis* expressing the OsARM1-GFP fusion protein were examined by confocal microscopy. Before confocal observation, the *Arabidopsis* leaves were infiltrated with PBS (pH 7.4) containing 2 ng/μL DAPI for 10 min. Bars = 20 μm. OsARM1-GFP was predominantly present in the nuclei (bottom images), while the GFP vector control was present in the nuclei and cytosol (top images).

To determine the subcellular localization of OsARM1, we cloned *OsARM1* in the pBI-eGFP vector (Xiao et al., 2008) fused to the N-terminus of the gene encoding enhanced green fluorescent protein (eGFP). We then transiently co-expressed the empty vector pBI-eGFP (GFP) or the construct expressing OsARM1-GFP together with the nuclear marker AtARF4-RFP in isolated rice protoplasts and detected the fluorescence by confocal laser scanning microscopy. In the rice protoplasts expressing just GFP, the green fluorescent signals were observed throughout the cell; by contrast, the green fluorescent signals of OsARM1-GFP were primarily detected in the nucleus, with weak signals in the cytosol (Figure 3A). The nuclear localization of OsARM1-GFP was further confirmed its co-localization with the red fluorescent signal of the nuclear marker AtARF4-RFP (Figure 3A; Piya et al., 2014). To confirm this observation, we also transformed the OsARM1-GFP construct into wild-type *Arabidopsis* (Col-0) to generate transgenic lines. In contrast to transgenic lines expressing GFP from the empty vector, the OsARM1-GFP fusion protein predominately localized to the nucleus in the *Arabidopsis* leaf epidermal cells (Figure 3B). To further confirm this observation, we stained leaf cells of *OsARM1-GFP* plants with 4′,6-diamidino-2-phenylindole (DAPI) and found that OsARM1-GFP co-localized with DAPI, which confirmed the nuclear localization of OsARM1 in *Arabidopsis* cells (Figure 3B; bottom images).

### Knockout of *OsARM1* increases rice tolerance to arsenic stress

**Figure 4:**
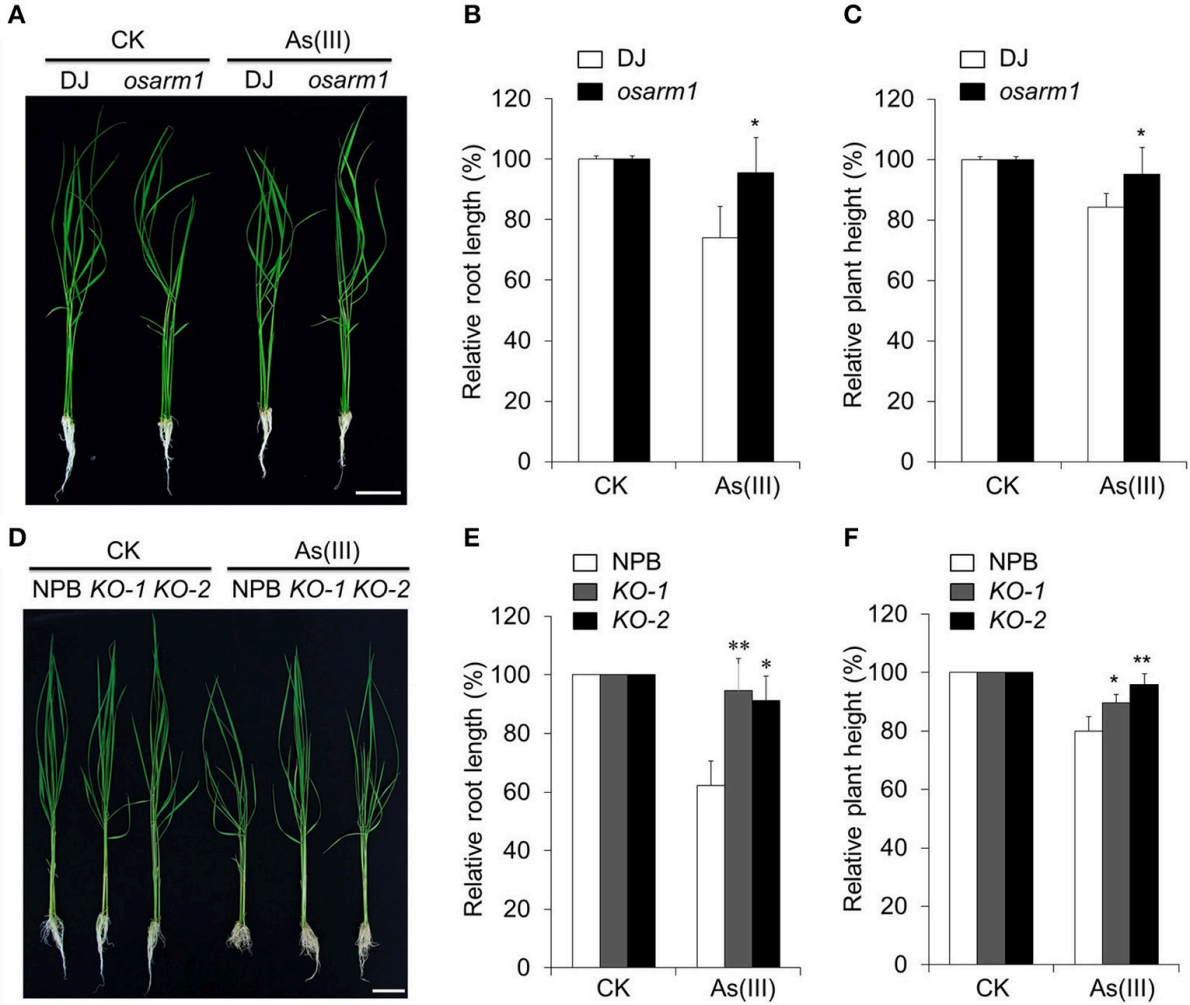
Knockout of *OsARM1* confers enhanced tolerance to As(III)-induced stress. **(A)** Phenotypes of 2-week-old wild-type DJ and *osarm1* plants grown on Kimura B nutrient solution without (CK) or with 40 μM As(III) for 7 days. **(B,C)** Relative root elongation **(B)** and relative plant height **(C)** of DJ and *osarm1* plants after As(III) treatment in **(A)**. **(D)** Phenotypes of 2-week-old wild-type Nipponbare (NPB) and *OsARM1-KO* transgenic lines (*KO-1* and *KO-2*) grown on Kimura B nutrient solution without (CK) or with 40 μM As(III) for 7 days. **(E,F)** Relative root elongation **(E)** and plant height **(F)** of Nipponbare and *OsARM1-KO*s (*KO-1* and *KO-2*) after As(III) treatment in **(D)**. The experiments were repeated three times (biological replicates), and >10 plants were used for each genotype in a single experiment. Data are means ± *SD* (*n* = 30) calculated from three independent experiments. Asterisks indicate significant differences from wild type (^*^P < 0.05; ^**^P < 0.01 by Student's *t*-test). Bars = 4 cm.

To assess the role of OsARM1 in the As(III) response, we identified a rice T-DNA insertion mutant, *osarm1* (PFG_3A-12233.R), from the Rice T-DNA Insertion Sequence Database (Jeon et al., 2000; Jeong et al., 2006). Genotyping and sequence analyses demonstrated that a T-DNA insertion is located in the promoter region (-449) of *OsARM1* in this mutant (Figures S2A,B). RT-PCR analysis revealed that the expression of *OsARM1* was clearly reduced in the *osarm1* homozygous mutant in various tissues compared to the wild-type control (Figure S2C). When the seedlings were exposed to 40 μM As(III) for 7 days, the root length and plant height of DJ were significantly reduced (Figures 4A–C). By contrast, the *osarm1* mutant showed increased tolerance to As(III) stress, including increased plant height and root length, compared to the wild-type control (Figure 4A; Figure S10). The enhanced As(III) tolerance of *osarm1* mutants was further confirmed by calculating the relative root elongation and plant height of rice plants before and after As(III) treatment (Figures 4B,C).

Phenotypically, young *osarm1* seedlings showed no significant morphological changes; however, mature *osarm1* plants were semi-dwarf and partially sterile compared to wild-type (DJ) plants grown under the same conditions (Figures S3A,B). As a result, the seed setting rate of *osarm1* was only approximately 30% that of DJ (Figure S3C). To test whether the growth inhibition and reduced fertility of the *osarm1* mutant during late development resulted from the lesion in *OsARM1* (Figure S3), we used CRISPR-Cas9 to generate rice lines with knockout mutations of *OsARM1* (designated *OsARM1-KOs*). PCR and sequencing identified two alleles: a 1-bp insertion in target I and a 1-bp deletion in target II, among the transgenic lines (Figure S4). Such mutations would cause a frameshift in the ORF, leading to early termination of translation of OsARM1 protein. Further, the OsARM1 protein in the *OsARM1-KO* mutants was potentially to lack the intact structure and normal transactivation activity. The growth and fertility of both *OsARM1-KO* lines (*KO-1* and *KO-2*) were not obviously different from wild type (Figure 4D; Figures S3D–F), indicating that these phenotypes of *osarm1* likely result from a separate mutation, possibly caused by an unlinked T-DNA insertion. However, when 2-week-old plants were exposed to 40 μM As(III) for 7 days, *OsARM1-KOs* were more resistant to As(III) that the wild-type (NPB) control (Figure 4D). This phenotype is consistent with that of the *osarm1* T-DNA insertion mutant (Figure 4A). We calculated the relative root length and plant height after As(III) treatment, finding that the values for the *OsARM1-KO* plants were significantly higher than those of wild type (Figures 4E,F). In addition, when we reduced the concentration of As(III) to 2 and 5 μM, we observed no obvious inhibition of the root growth of *osarm1* and *OsARM1-KO* plants. However, the relative root lengths of wild type DJ and NPB were shorter than those of *osarm1* and *OsARM1-KO* plants (Figures S5A,B,D,E). Under such low As(III) concentrations, the relative heights of wild-type plants and the *OsARM1* mutants showed no significant differences (Figures S5C,F).

### Overexpression of *OsARM1* confers increased As(III) sensitivity in transgenic rice

**Figure 5:**
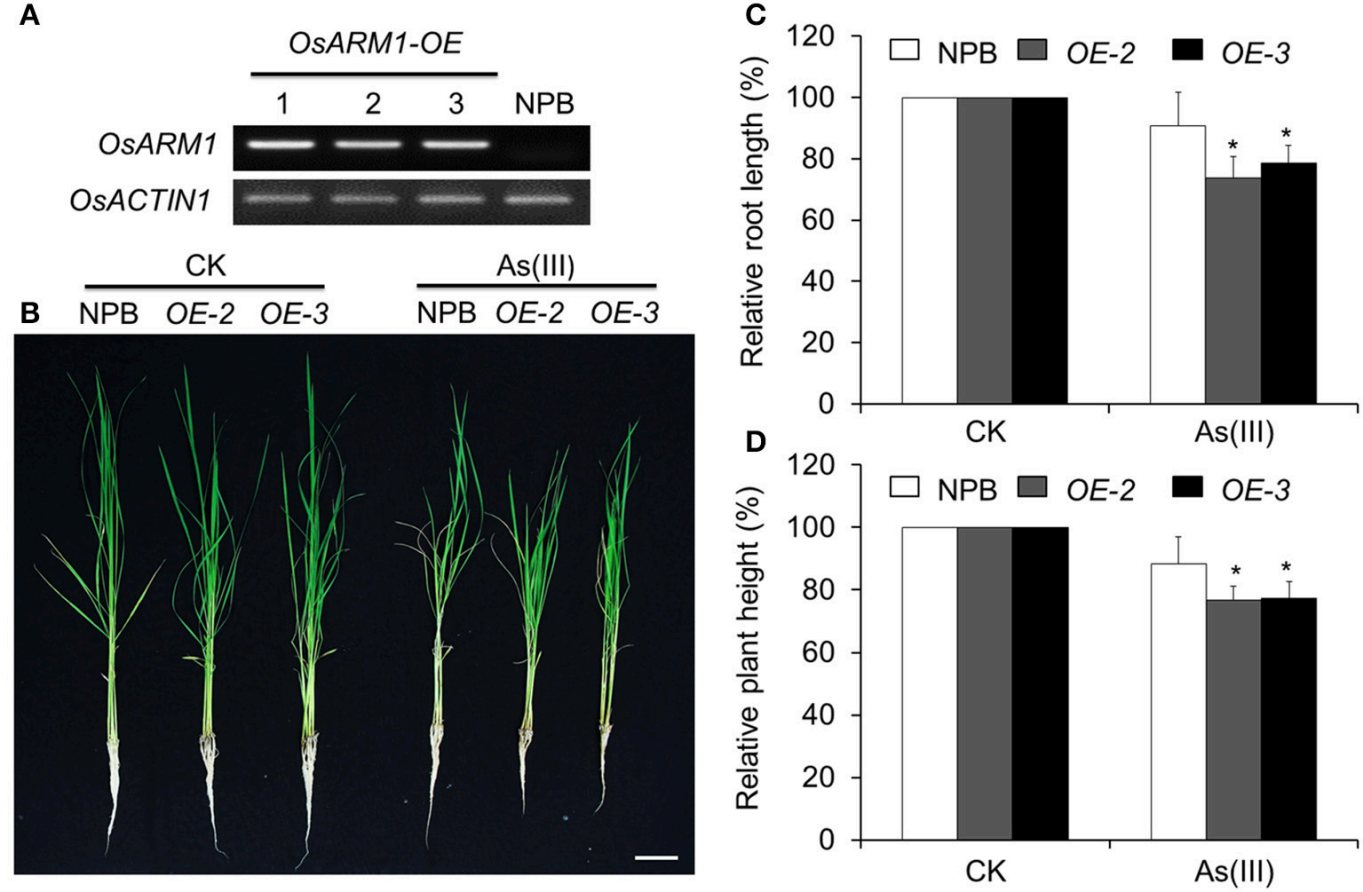
Overexpression of *OsARM1* attenuates tolerance to As(III). **(A)** The expression levels of *OsARM1* in the *OsARM1-OE* transgenic lines were determined by RT-PCR analysis. *OsACTIN1* was used as a reference gene. **(B)** Images of wild-type NPB and *OsARM1-OE* transgenic lines (*OE-2* and *OE-3*) treated without (CK) or with As(III). Two-week-old seedlings were grown on Kimura B medium or Kimura B medium containing 25 μM As(III) for 7 days. Bars = 4 cm. **(C,D)** Relative root elongation **(C)** and relative plant height **(D)** of NPB and *OsARM1-OE* transgenic lines (*OE-2* and *OE-3*) after As(III) treatment. The experiments were repeated three times (biological replicates), and >10 plants were used for each genotype in a single experiment. Data are means ± *SD* (*n* = 30) calculated from three independent experiments. Asterisks indicate significant differences from wild type (^*^P < 0.05 by Student's *t*-test).

To further evaluate the role of OsARM1 in plant As(III) tolerance, we generated transgenic lines overexpressing *OsARM1* via *Agrobacterium*-mediated transformation of wild-type plants (NPB). To this end, we cloned *OsARM1* into the binary vector pCXSN-Myc, under the control of the 35S promoter. RT-PCR analyses showed that *OsARM1* was overexpressed in the three independent *OsARM1-OE* T_0_ transgenic lines obtained (Figure 5A).

As shown in Figure 5, the shoot and root growth in lines *OE-2* and *OE-3* were not significantly different from that of wild type under normal growth conditions. After treatment with 25 μM As(III) for 7 days, the growth of both *OE* lines (*OE-2* and *OE-3*) lagged behind that of wild-type (NPB) seedlings (Figure 5B), indicating their increased sensitivity to As(III)-induced stress compared to NPB. Statistical analysis (Figure 5C) revealed that the root lengths of the two *OE* lines (*OE-2* and *OE-3*) grown on As(III)-containing liquid medium were 73.7 ± 7.0 and 78.6 ± 5.8%, respectively, of the lengths of *OE* roots not treated with As, and were significantly shorter than the NPB roots on As(III) medium (90.7 ± 11.1%). In the presence of 25 μM As(III) on day 7, the relative shoot heights of both *OE* lines (*OE-2* and *OE-3*) were significantly reduced compared to that of NPB (Figure 5D). Similarly, the root growth of the *OsARM1-OE* lines was suppressed by growth on 2 and 5 μM As(III) for 14 days (Figure S6). These findings imply that OsARM1 is involved in the regulation of the response to As(III) stress in rice.

### *OsARM1-KOs* and *OsARM1-OEs* show altered root-to-shoot translocation of As

**Figure 6:**
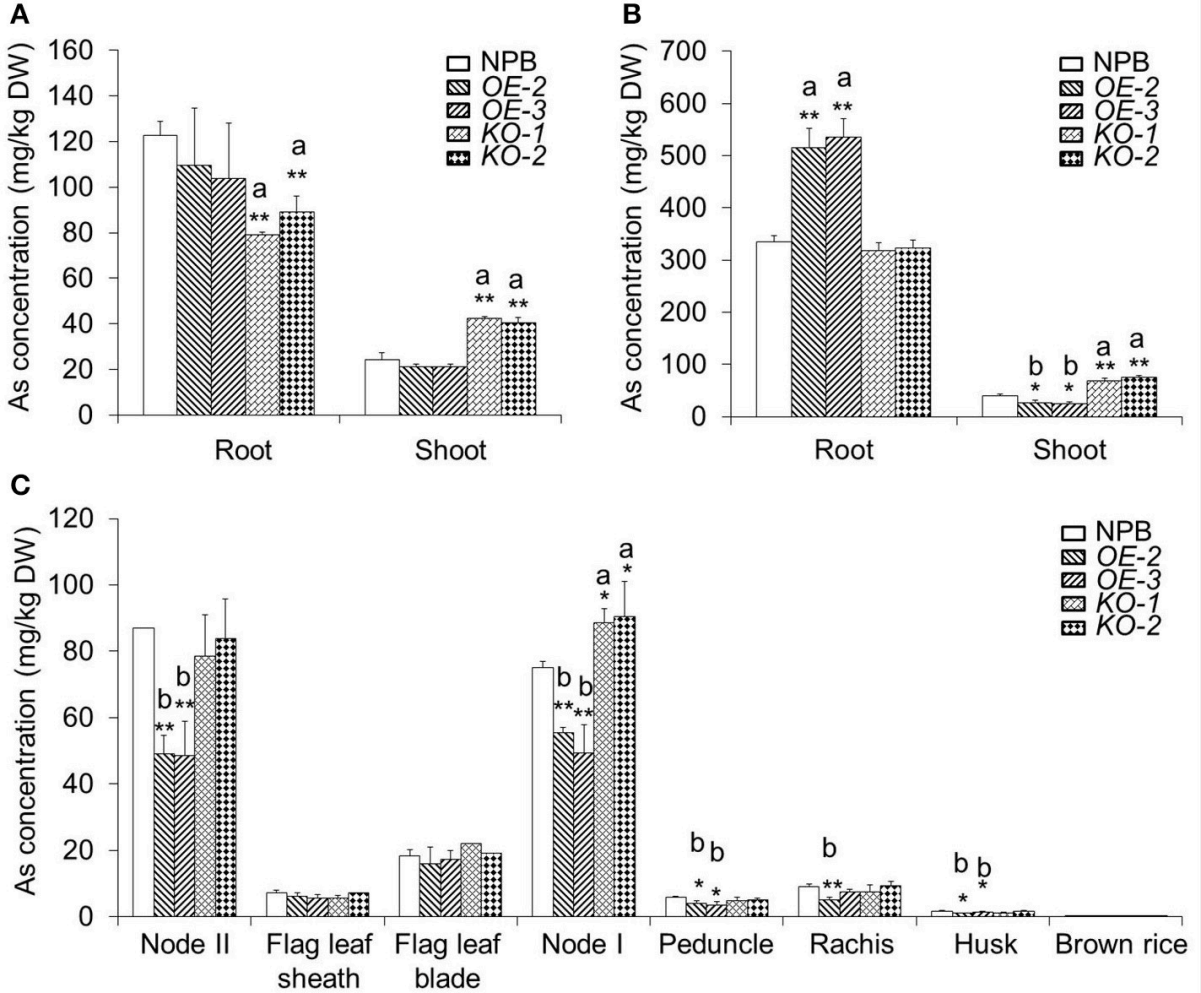
The As contents in various organs of wild-type NPB, *OsARM1-KOs*, and *OsARM1-OEs*. **(A,B)** The As levels in roots and shoots of Nipponbare, *OsARM1-KOs*, and *OsARM1-OEs*. Two-week-old wild-type NPB, *OsARM1-KOs* (*KO-1* and *KO-2*), and *OsARM1-KOs* (*OE-2* and *OE-3*) seedlings were exposed to Kimura B nutrient solution containing 2 μM **(A)** or 25 μM As(III) **(B)** for 7 days. The root and shoot samples were harvested separately for As determinations by atomic fluorescence spectrometry. **(C)** As contents in different organs in the aboveground parts of wild type NPB, *OsARM1-KOs* (*KO-1* and *KO-2*), and *OsARM1-KOs* (*OE-2* and *OE-3*) in As-containing soil (20 mg As per 1 kg soil) until the ripening stage. The organs were sampled and subjected to As determination by atomic fluorescence spectrometry. The experiments were repeated three times and the average data are shown. Data are means ± *SD* (*n* = 3) calculated from three independent experiments. Asterisks indicate significant differences from wild type (^*^P < 0.05; ^**^P < 0.01 by Student's *t*-test). “a” and “b” indicate values that are significantly higher or lower, respectively, in the transgenic lines than in wild type.

The increased and attenuated As(III)-tolerant phenotypes in *OsARM1-KOs* and *OsARM1-OEs*, respectively, may be due to altered uptake or translocation of As. To investigate this, we measured the As contents in wild-type (NPB), *OsARM1-KOs* (*KO-1* and *KO-2*), and *OsARM1-OEs* (*OE-2* and *OE-3*) plants. After exposure to 2 μM As(III) for 7 days, the root and shoot samples were harvested separately for As measurements. As shown in Figure 6A, even though the *OsARM1-OEs* and NPB showed no significant differences in As contents of their roots, the As levels in roots of *OsARM1-KOs* were lower than the wild-type NPB, and the As levels in shoots of *OsARM1-KOs* were higher than NPB, while the As content in shoots of *OsARM1-OEs* were a little lower than NPB. Moreover, in plants grown on 25 μM As(III) for 7 days, the roots of *OE-2* and *OE-3* had As levels that were 1.5- and 1.6-fold higher, respectively, than that of wild type. In contrast to the results in roots, the As contents in *OsARM1-OE* shoots were lower than in NPB and As contents in *OsARM1-KO* shoots (*KO-1* and *KO-2*) were 1.7- and 1.9-fold higher, respectively, than that of NPB. No significant differences of As contents were detected in root tissues between the *OsARM1-KO* lines and NPB (Figure 6B), but the absolute values of As concentrations were lower in the *OsARM1-KOs* (317.3 ± 16.4 and 323.4 ± 15.7 mg/kg DW) in the roots in comparison with wild type (334.1 ± 12.7 mg/kg DW). These results indicate that OsARM1 is likely involved in regulating uptake and root-to-shoot translocation of As in rice.

To assess the distribution of As in shoots, we measured the As concentrations in various organs above node II in NPB, *OsARM1-KOs*, and *OsARM1-OEs* plants. All plants were grown in 20 mg As per kg soil until the ripening stage. Compared with wild-type NPB, the *OsARM1-KO* lines contained significantly higher levels of As in node I (Figure 6C). By contrast, the As contents were reduced in nodes I and II of the *OsARM1-OE* lines compared to wild type (Figure 6C). As a result, the *OsARM1-OEs* showed slightly reduced levels of As in the peduncle, rachis, and husk (Figure 6C). Taken together, the increased and reduced accumulation of As in node I of *OsARM1-KOs* and *OsARM1-OEs*, respectively, relative to wild type suggest that OsARM1 may function in regulating the expression of key arsenic transporter genes in this tissue, a vital site for controlling As translocation to the grain.

### Expression of transporter genes involved in As uptake and translocation is regulated by OsARM1 under As(III) stress

**Figure 7:**
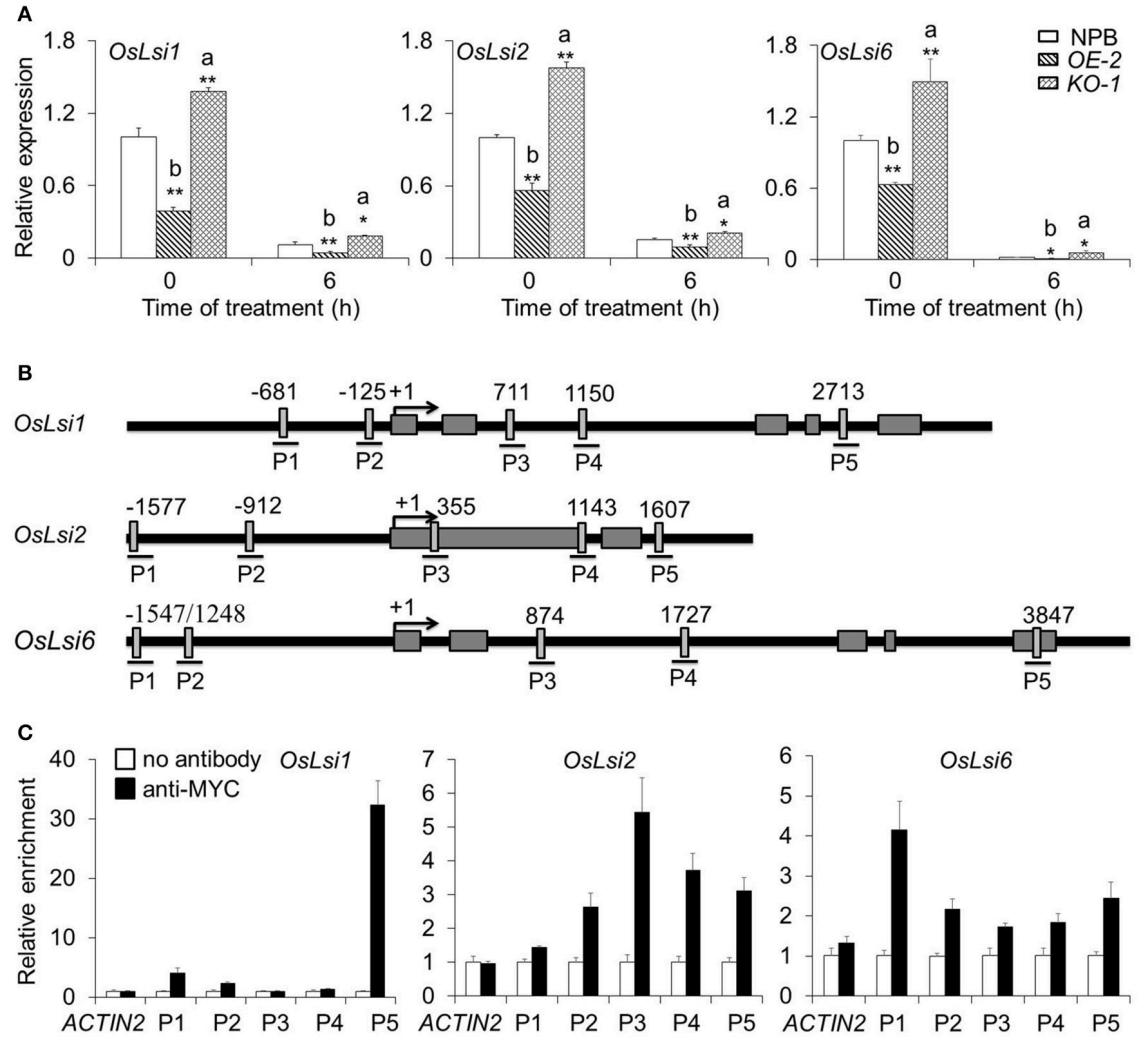
OsARM1 directly interacts with *OsLsi1, OsLsi2*, and *OsLsi6* in rice. **(A)** Expression of As transporter genes in wild-type NPB, *OsARM1-KO*, and *OsARM1-OE*. Total RNA was extracted from the roots and shoots of 2-week-old seedlings (wild-type NPB, *OE-2*, and *KO-1*) treated with Kimura B nutrient solution containing 50 μM As(III) for 0 and 6 h. The expression levels of *OsLsi1, OsLsi2*, and *OsLsi6* in roots were examined by qRT-PCR analyses. *OsGAPDH* was used as a reference gene. The experiment was repeated three times with similar results. Asterisks indicate significant differences from wild type (^*^P < 0.05; ^**^P < 0.01 by Student's *t*-test). “a” and “b” indicate values that are significantly higher or lower, respectively, in *OE-2* or *KO-1* than in wild type. **(B)** Schematic diagram of the potential AC-I element (ACC(A/T)A(A/C)) in the promoter and genomic sequences of *OsLsi1, OsLsi2*, and *OsLsi6*. Lines under the boxes indicate sequences detected by ChIP-qPCR assays. Numbers indicate the nucleotide positions relative to the corresponding translational start site (ATG), which is shown as +1. **(C)** ChIP-qPCR analyses showing the *in vivo* interaction between OsARM1 and the predicted AC-I element in the promoters of *OsLsi1, OsLsi2*, and *OsLsi6*. Protein/DNA complexes isolated from the root of *OsARM1-OE* (*OE-2*) transgenic rice were immunoprecipitated with or without the anti-Myc antibody, respectively. For each promoter, various DNA fragments were used to determine the enrichment of the DNA fragment containing the AC-I element. A promoter fragment of *OsACTIN1* was used as a negative control. The experiment was repeated three times with similar results.

To identify differentially expressed genes involved in As translocation under As(III) stress, we extracted total RNA from roots of 2-week-old wild-type NPB, *OsARM1-KO*, and *OsARM1-OE* seedlings under normal conditions or seedlings treated for 6 h with Kimura B nutrient solution containing 50 μM As(III). We analyzed the expression of representative As-responsive transporter genes, *OsLsi1, OsLsi2*, and *OsLsi6*, by qRT-PCR. As expected, under As(III) stress, the transcripts of *OsLsi1, OsLsi2*, and *OsLsi6* were upregulated in *OsARM1-KO* lines but downregulated in *OsARM1-OE* lines compared with wild-type (NPB) plants (Figure 7A).

The altered expression pattern of *OsLsi1, OsLsi2*, and *OsLsi6* in the *OsARM1-KO* and *OsARM1-OE* lines suggested that OsARM1 regulates the plant response to As(III) by modulating the transcript levels of these transporters. To investigate the direct regulation of these As transporter genes by the OsARM1 transcription factor, we used the *OsARM1-OE* lines (which express an ARM1-Myc fusion protein) for further chromatin immunoprecipitation-quantitative PCR (ChIP–qPCR) analysis. Given that R2R3-MYB transcription factors bind to the AC-I element [ACC(A/T)A(A/C)] in the promoters and genomes of target genes to directly regulate their transcription (Zhong and Ye, 2012), we first analyzed the promoter sequences of *OsLsi1, OsLsi2*, and *OsLsi6*. Five (P1, P2, P3, P4, and P5) AC-I elements were detected in the *OsLsi1, OsLsi2*, and *OsLsi6* sequences (Figure 7B). ChIP-qPCR data revealed that for each *OsLsi* gene, only one DNA fragment, P5 from *OsLsi1*, P3 from *OsLsi2*, and P1 from *OsLsi6*, was enriched in the DNA immunoprecipitates produced using anti-Myc antibody (Figure 7C). To further determine the interaction between OsARM1 and As transporter genes, we took advantage of the two available transgenic *Arabidopsis* lines expressing OsARM1-GFP, *GFP-2*, and *GFP-9* (Figure S7A). Phenotypic analyses (Figures S7B,C) showed that both transgenic lines were highly sensitive to As(III) treatment compared to wild type (Col-0), with responses resembling that of the *OsARM1-OE* lines (Figure 5), suggesting that OsARM1-GFP is functional in *Arabidopsis*.

We also analyzed the expression of *AtNIP1;1, AtNIP3;1*, and *AtNIP5;1*, three well-known *Arabidopsis* genes homologous to aquaporin, under As(III) stress (Ali et al., 2009; Kamiya et al., 2009; Xu et al., 2015). The expression of *AtNIP3;1* and *AtNIP5;1* declined after treatment with 40 μM As(III) for 6 h and their expression levels were higher in *GFP-9* than in wild-type Col-0. At the same time, the expression of *AtNIP1;1* increased in *GFP-9* after As(III) treatment (Figure S8A). Promoter sequence analysis identified three AC-I elements (P1, P2, and P3) in the promoter regions of *AtNIP1;1, AtNIP3;1*, and *AtNIP5;1* (Figure S8B). ChIP-qPCR data showed that one or two DNA fragments from each promoter, i.e., P1 in *AtNIP1;1*, P3 in *AtNIP3;1*, and P1 and P2 in *AtNIP5;1*, were enriched in the DNA immunoprecipitates produced using anti-GFP antibody (Figure S8C). These results confirm that OsARM1 can directly bind to the promoter regions of *OsLsi1, OsLsi2*, and *OsLsi6* in rice as well as *AtNIP1;1, AtNIP3;1*, and *AtNIP5;1* in *Arabidopsis*.

## Discussion

As(III) is an inorganic species of As that causes prolonged, toxic effects on human and plant health. Development crop cultivars, particularly rice, with the ability to tolerate high levels of As with minimal accumulation of As in their edible parts may help protect people from As poisoning. Recent findings suggest that phloem transport accounts for ~90% of As(III) accumulation in rice grains (Carey et al., 2010). Understanding the mechanism of As transport represents an initial step in reducing As contents in rice grains. Increasing evidence suggests that numerous rice As transporters play pivotal roles in As uptake, long-distance translocation, and detoxification (Song et al., 2014; Li et al., 2016). However, how these transporters are transcriptionally regulated under As stress remains unclear. In this study, we identified OsARM1, an R2R3 MYB transcription factor that is responsible for the transcriptional regulation of transporter genes involved in As uptake and translocation under As stress.

Our results show that, based on the relative root elongation and shoot height, the *OsARM1* T-DNA insertion mutant (*osarm1*) and site-specific knockout mutants *OsARM1-KOs* grew better than wild type under As treatment (Figure 4; Figure S5), suggesting that *OsARM1* mutants are more tolerant to arsenic stress. By contrast, the *OsARM1-OE* lines displayed reduced relative root elongation and shoot height compared with wild type (Figure 5; Figure S6), implying the involvement of OsARM1 in the regulation of As responses in rice. It is noteworthy that when we tested their sensitivities to As(III) stress, the *osarm1* mutant and *OsARM1-KO* lines showed consistent tolerance to As(III) exposure (Figure 4; Figure S5). However, we observed that the *osarm1* mutant was semi-dwarf and partially sterile (Figures S3A–C), phenotypes that were not exhibited by the *OsARM1-KO* lines (Figures S3D–F). As both of the *OsARM1-KO* lines were normal in growth and fertility (Figures S3D–F), these results suggest that the reduced fertility phenotype in the *osarm1* mutant is likely caused by an extra T-DNA insertion in another gene related to growth and fertility, rather than the *OsARM1* mutation. This possibility has also been demonstrated in a previous study with regard to the T-DNA insertional mutagenesis for functional genomics in rice, which showed that about 65% of lines contained more than one T-DNA insertion (Jeon et al., 2000). Therefore, we used the *OsARM1-KO-1* and *KO-2* lines, instead of the *osarm1* mutant, for the functional characterization of OsARM1.

Transcriptional regulation of plant responses to heavy metals has long been investigated (Quinn and Merchant, 1995). Although early studies identified numerous *cis*-elements that function in the responses to various heavy metal stresses, such as cadmium and copper stress (Quinn and Merchant, 1995; Kusaba et al., 1996; Nagae et al., 2008), their corresponding DNA-binding proteins have not been identified. Compared to transporters, transcription factors involved in heavy metal stress have received much attention in the past few years (Yu et al., 2012; Gao et al., 2015). Several transcription factors from different plant species that function in the response to cadmium have been characterized using forward or reverse genetic approaches (Sun et al., 2015; Chen et al., 2016; Yang et al., 2016).

In rice plants, nodes are pivotal tissues for As storage and uploading to grains (Chen et al., 2015). Under anaerobic conditions such as flooded paddy soil, As(III) is predominately taken up by aquaporin channels in plants (Zhao et al., 2009). Moreover, nodes contain a sophisticated vascular system to regulate long-distance translocation of nutrients to storage organs during grain filling (Yamaji and Ma, 2014). During this process, several transporter proteins, including Lsi1 and Lsi2, are involved in uptake and transport of As from the outside medium to rice grains (Ma et al., 2008; Chen et al., 2015). Lsi1 and Lsi2 accumulate in rice roots and serve as major transporters that are essential for As(III) uptake or root-to-shoot transport (Ma et al., 2008). By contrast, the C-type ABC transporter OsABCC1 helps limit As allocation from the upper node to the grain, which represents a useful strategy for reducing As accumulation in rice grains (Song et al., 2014). Previous (Yu et al., 2012) and current findings (Figure 7A) indicate that *Lsi1, Lsi2*, and *Lsi6* are downregulated by As(III) treatment in roots. By contrast, *OsABCC1* is significantly upregulated in response to high levels (5 μM) of As, although its expression level is unaffected at lower concentrations (0.5 μM) of As (Song et al., 2014). These results indicated that in response to As stress, vascular system-localized As-responsive transporters are transcriptionally regulated by upstream transcription factors.

The expression of *OsARM1* was particularly higher in spikelet than in nodes (node II and node I) in the qRT-PCR data (Figure 1A). However, our results from GUS staining of plants carrying a promoter-GUS construct (*OsARM1pro::GUS*) showed that the MYB-type transcription factor gene *OsARM1* was specifically expressed in vascular bundles of various rice tissues (Figure 2), with GUS expression concentrated in the nodes (nodes I and II, Figure 2). This difference may due to the usage of hand-cut cross-sections for the node tissues which is better for infiltration of GUS staining solution into enlarged vascular bundles, compared to the intact tissues of husk and anther. Alternatively, the use of 1.5-Kb promoter sequence for the OsARM1_pro_::GUS construction may loss the specific functional elements in the introns or exons of *OsARM1* genes and subsequently lead to the difference in the data of qRT-PCR and GUS assays. Promoter analysis using the fragment upstream of the start codon has proven to be workable in several other studies (e.g., Zhang et al., 2013; Song et al., 2014). Therefore, we generated the transgenic lines expressing *OsARM1pro::GUS* as a general way to investigate the expression of *OsARM1* in the initial stage of this study. Indeed, our results indicated that *OsARM1* expression was inducible by As(III) treatment (Figures 2B, panels e–h), suggesting the existence of As(III)-responsive regulatory elements in the *OsARM1* promoter. Nonetheless, we cannot exclude the possible involvement of introns or exons in the regulation of *OsARM1* expression. Given that intron sequences have been extensively studied in animals and plants and play important regulatory roles in gene expression (Rose et al., 2008), future investigation of the conserved elements in the promoter, introns, and exons of *OsARM1* will deepen our understanding of the mechanisms regulating this gene.

To investigate this hypothesis, we measured total As contents in various organs of *OsARM1-OEs, OsARM1-KOs*, and wild-type plants (Figure 6). Analyses of As contents in roots and shoots suggested that the *OsARM1-OE* plants accumulated much more As in roots, but transported less As to shoots compared to wild type, whereas the *OsARM1-KOs* translocated more As to shoots (Figure 6B), suggesting that OsARM1 plays a negative role in root-to-shoot translocation of As. Among the parts of the shoot, the *OsARM1-KO* lines accumulated much more As in node I, whereas the *OsARM1-OE* lines accumulated less As in nodes I and II compared to wild type (Figure 6C). Moreover, unlike the *osabcc1* mutant, which accumulates more As in node I and less As in the grain compared to wild type (Song et al., 2014), the *OsARM1-KOs* and wild type showed no significant differences in As contents in the organs above node I, especially in grains (Figure 6C). These findings suggest that additional transcription factors may share redundant functions with OsARM1.

Further transcriptional analysis showed that several rice transporter genes, including *OsLsi1, OsLsi2*, and *OsLsi6*, were differentially expressed in *OsARM1-OEs, OsARM1-KOs*, and wild-type plants (Figure 7A). Previous studies revealed that the transporters OsLsi1, OsLsi2 and OsLsi6 have different functions in the plant response to As stress. As(III) is taken up by rice roots through Lsi1 and effluxed toward the stele for xylem loading by Lsi2 (Ma et al., 2006, 2007). However, Lsi6 transports Si out of the xylem and affects Si distribution in rice shoots (Yamaji et al., 2008). Even though Lsi6 does not contribute substantially to As uptake by rice roots, it has As transport activity when expressed in oocytes (Ma et al., 2008). Our results indicate that more As may be assimilated by *OsARM1-KOs* plants than *OsARM1-OEs* plants. However, we detected a higher As concentration in the root of *OsARM1-OEs* (Figure 6B). In fact, less As was transported to shoots of *OsARM1-OEs*, which might contribute to higher As concentration in the roots (Figure 6B). It is also possible that the roots of *OsARM1-OEs* were damaged by high As concentrations, leading to increased sensitivity to As stress (Figures 5B–D). These data demonstrate that OsARM1 plays an important role in root-to-shoot translocation, rather than the uptake of As by roots. In addition, we found that the expression levels of several ABC transporters (*OsABCG41, OsABCB11*, and *OsABCC14*) were significantly changed in the *OsARM1-KOs* or *OsARM1-OEs* (Figure S9), indicating that OsARM1 may function as an upstream regulator involved in modulation of As uptake and root-to-shoot translocation. Future work, such as searching for other direct target transporter genes of OsARM1, may help in explaining the complexity of OsARM1-associated phenotypes.

ChIP-qPCR analysis provided direct evidence of the interaction between OsARM1 and the 5'-flanking or genomic regions of *OsLsi1, OsLsi2*, and *OsLsi6* in rice (Figure 7C), as well as that of *AtNIP1;1, AtNIP3;1*, and *AtNIP5;1* in *Arabidopsis* (Figure S8C), suggesting that OsARM1 may regulate the uptake and root-to-shoot translocation of As by directly suppressing the expression of the NIP-encoding genes *OsLsi1, OsLsi2*, and *OsLsi6*. We further analyzed the sequences of *OsLis1*-P5, *OsLsi2*-P3, and *OsLsi6*-P1 and found that the R2R3-MYB transcription factor binding motif, the AC-I element [ACC(A/T)A(A/C)], was conserved in all of these fragments. As Lsi2-P3 was located in the exon, we checked previous studies about the functionality of regulatory sequences in the exon. Interestingly, ChIP-qPCR analysis results from a more recent study (Wang et al., 2017) reported that in *Arabidopsis*, the transcription factor MYC2 bind to the regions including the G-box located in the exon of the target gene *FLOWERING LOCUS T*. These findings suggest the potential role of exon sequences in transcriptional regulation of gene expression, although this is not common and the underlying mechanism remains to be further investigated.

In conclusion, OsARM1 attenuates the translocation of As from roots to shoots, thereby representing an important component in the plant response to As stress. In particular, OsARM1 may regulate the transcript levels of rice ABC transporter genes by directly binding to their promoter regions. The localization of OsARM1 to the vascular system is an efficient way to prevent the translocation of As to aboveground tissues. This discovery sheds light on the transcriptional regulation of As translocation and will facilitate the development of strategies for engineering rice with decreased As levels in grain in the future. Further investigation of the regulatory networks of OsARM1 using transcriptome technology and functional characterization of the protein interactors of OsARM1, as well as the genetic linkages between OsARM1 and As-responsive transporters, will increase our understanding of the role of OsARM1 in the plant response to As(III) stress.

## Author contributions

QC and SX designed the study. FW, MC, LJY, LX, LBY, HQ, MX, WG, and ZC carried out the experiments. FW, KY, JZ, RQ, WS, QC, and SX analyzed the data. FW, SX, and QC wrote the manuscript.

### Conflict of interest statement

The authors declare that the research was conducted in the absence of any commercial or financial relationships that could be construed as a potential conflict of interest.

## Supplementary material

Figure S1 Expression of *OsARM1*_*pro*_*::GUS* in the vascular cells of roots, leaves, and stems after As(III) treatment. **(A,B)** Transverse sections of roots **(A)** and leaves **(B)** showing GUS staining in the vascular cells. **(C,D)** Images showing the GUS staining in the epidermis of stems **(C)** and leaves **(D)**, respectively. Bars = 20 μm.

Figure S2 Identification of the *osarm1* T-DNA insertion mutant. **(A)** Location of T-DNA insertion site in *OsARM1* in the *osarm1* mutant (Os05g37060). Primer pairs XS830/XS831, XS830/PGVRB, and XS831/PGVRB were used to genotype the mutant. Primer pair XS147/XS148 was used to determine *OsARM1* expression levels. E1 and E2 denote exon 1 and exon 2, respectively. **(B)** Genotyping analysis of *osarm1* mutant by tri-primer PCR. All five independent plants are homozygous *osarm1* mutants. **(C)** RT-PCR showing the expression levels of *OsARM1* in various organs of wild type (DJ) and *osarm1*. *OsACTIN1* was used as a reference gene. P, panicle; G, grain; R, root; S, stem; L, leaf blade.

Figure S3 Phenotypes of *OsARM1* mutants. The *osarm1* seedlings showed reduced plant height **(A)**, panicle number **(B)**, and seed setting rate **(C)** compared to the wild-type DJ. But all the phenotypes mentioned above of *OsARM1-KO* lines were the same as wild-type NPB **(D–F)**. Asterisks indicate significant differences from wild type (^**^*P* < 0.01 by Student's *t*-test).

Figure S4 Generation of *OsARM1-KO* transgenic lines. **(A)** Schematic diagram of the vector *OsARM1* T_1_T_2_-*Cas9* (*OsARM1*-*KO*). **(B)** Genome editing efficiency of *OsARM1-KO* transgenic rice lines. Two independent transgenic lines containing a 1-bp insertion and 1-bp deletion, respectively, at the expected cleavage site were obtained.

Figure S5 Knockout of *OsARM1* confers enhanced tolerance to low concentrations of As(III). **(A,D)** Phenotypes of 2-week-old wild-type DJ and *osarm1* or wild-type NPB and *OsARM1-KO* transgenic lines (*KO-1* and *KO-2*) plants grown on Kimura B nutrient solution without (CK) or with 2 or 5 μM As(III) for 14 days. During the treatment, renewed the treatment solution on the 7th day. **(B,C)** Relative root elongation **(B)** and relative plant height **(C)** of DJ and *osarm1* plants after As(III) treatment in **(A)**. **(E,F)** Relative root elongation **(E)** and plant height **(F)** of NPB and *OsARM1-KO*s (*KO-1* and *KO-2*) after As(III) treatment in **(D)**. Asterisks indicate significant differences from wild type (^*^*P* < 0.05; ^**^*P* < 0.01 by Student's *t*-test). Bars = 4 cm.

Figure S6 Overexpression of *OsARM1* attenuates tolerance to low concentrations of As(III). **(A)** Images of wild-type NPB and *OsARM1-OE* transgenic lines (*OE-2* and *OE-3*) treated without (CK) or with As(III). Two-week-old seedlings were grown on Kimura B medium or Kimura B medium containing 2 or 5 μM As(III) for 14 days. During the treatment, renewed the treatment solution on the 7th day. Bars = 4 cm. **(B,C)** Relative root elongation **(B)** and relative plant height **(C)** of NPB and *OsARM1-OE* transgenic lines (*OE-2* and *OE-3*) after As(III) treatment. Asterisks indicate significant differences from wild type (^*^*P* < 0.05; ^**^*P* < 0.01 by Student's *t*-test).

Figure S7 Phenotypic analysis of transgenic *Arabidopsis* expressing OsARM1-GFP in response to As stress. **(A)** RT-PCR detected the expression level of *OsARM1* in 35S::*OsARM1-GFP* transgenic *Arabidopsis*. **(B)** Wild type (Col-0) and *OsARM1-GFP* transgenic lines (*GFP-2* and *GFP-9*) were germinated on 1/2 MS medium for 7 days. The seedlings were subsequently transferred to 1/2 MS medium (CK) or 1/2 MS medium containing 10 or 20 μM As(III) and grown vertically. The images were taken at 2 weeks after treatment, and the relative dry weights were calculated thereafter **(C)**. The experiments were repeated three times (biological replicates), and >10 plants were used for each genotype in a single experiment. Data are means ± *SD* (*n* = 3) of three biological replicates. Asterisks indicate significant differences from wild type (^**^*P* < 0.01 by Student's *t*-test).

Figure S8 OsARM1 directly interacts with the promoters of *AtNIP1;1, AtNIP3;1*, and *AtNIP5;1* in *Arabidopsis*. **(A)** Expression of As-related transporter genes in *Arabidopsis*. Total RNA was extracted from the wild-type Col-0 and *GFP-9* plants grown on half-strength MS medium for 14 d then transferred to filter paper soaked with 40 μM As(III) for 0 and 6 h. The expression levels of *AtNIP1;1, AtNIP3;1*, and *AtNIP5;1* were examined by qRT-PCR analyses. *AtACTIN2* was used as a reference gene. Asterisks indicate significant differences from wild type (^*^*P* < 0.05; ^**^*P* < 0.01 by Student's *t*-test). **(B)** Schematic diagram of the potential AC-I element (ACC(A/T)A(A/C)) in the promoter sequences of *AtNIP1;1, AtNIP3;1*, and *AtNIP5;1*. Lines under the boxes indicate sequences detected by ChIP-qPCR. Numbers indicate the nucleotide positions relative to the corresponding translational start site (ATG), which is shown as +1. **(C)** ChIP-qPCR analyses showing the *in vivo* interaction between OsARM1 and the predicted AC-I element in the promoters of *AtNIP1;1, AtNIP3;1*, and *AtNIP5;1*. Protein/DNA complexes isolated from the whole seedlings of *OsARM1-GFP* transgenic *Arabidopsis* were immunoprecipitated with or without the anti-GFP antibody. For each promoter, various DNA fragments were used to determine the enrichment of the DNA fragment containing the AC-I element. A promoter fragment of *AtACTIN2* was used as a negative control. The experiment was repeated three times with similar results.

Figure S9 Expression of As-responsive transporter genes in wild type, *OsARM1-KO*, and *OsARM1-OE*. Total RNA was extracted from the shoots of 2-week-old seedlings (wild-type NPB, *OE-2*, and *KO-1*) treated with Kimura B nutrient solution containing 50 μM As(III) for 0 and 6 h. The expression levels of *OsABCG41, OsABCB11*, and *OsABCC14* were examined by qRT-PCR analyses. *OsGAPDH* was used as a reference gene. The experiment was repeated three times with similar results. Asterisks indicate significant differences from wild type (^*^*P* < 0.05; ^**^*P* < 0.01 by Student's *t*-test). “a” and “b” indicate values that are significantly higher or lower, respectively, in *OE-2* or *KO-1* than in wild type.

Figure S10 Absolute values of root length **(A)** and plant height **(B)** of DJ and *osarm1* in Figure 4A. Two-week-old wild-type DJ and *osarm1* plants grown on Kimura B nutrient solution without (CK) or with 40 μM As(III) for 7 days. The experiment was repeated three times with similar results. Asterisks indicate significant differences from wild type DJ (^*^*P* < 0.05; ^**^*P* < 0.01 by Student's *t*-test).
