# Supplementary figures and images for: OsARM1, an R2R3 MYB Transcription Factor, Is Involved in Regulation of the Response to Arsenic Stress in Rice
Source: Front Plant Sci. 2017 Oct 30;8:1868. doi: 10.3389/fpls.2017.01868 (PMC5670359; doi:10.3389/fpls.2017.01868)

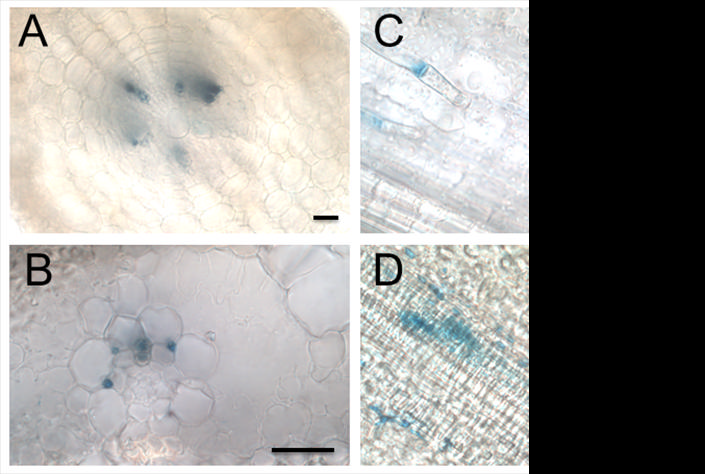

Supplement: Figure S1 — Expression of OsARM1pro::GUS in the vascular cells of roots, leaves, and stems after As(III) treatment. (A,B) Transverse sections of roots (A) and leaves (B) showing GUS staining in the vascular cells. (C,D) Images showing the GUS staining in the epidermis of stems (C) and leaves (D), respectively. Bars = 20 μm. [file Image1.TIF]

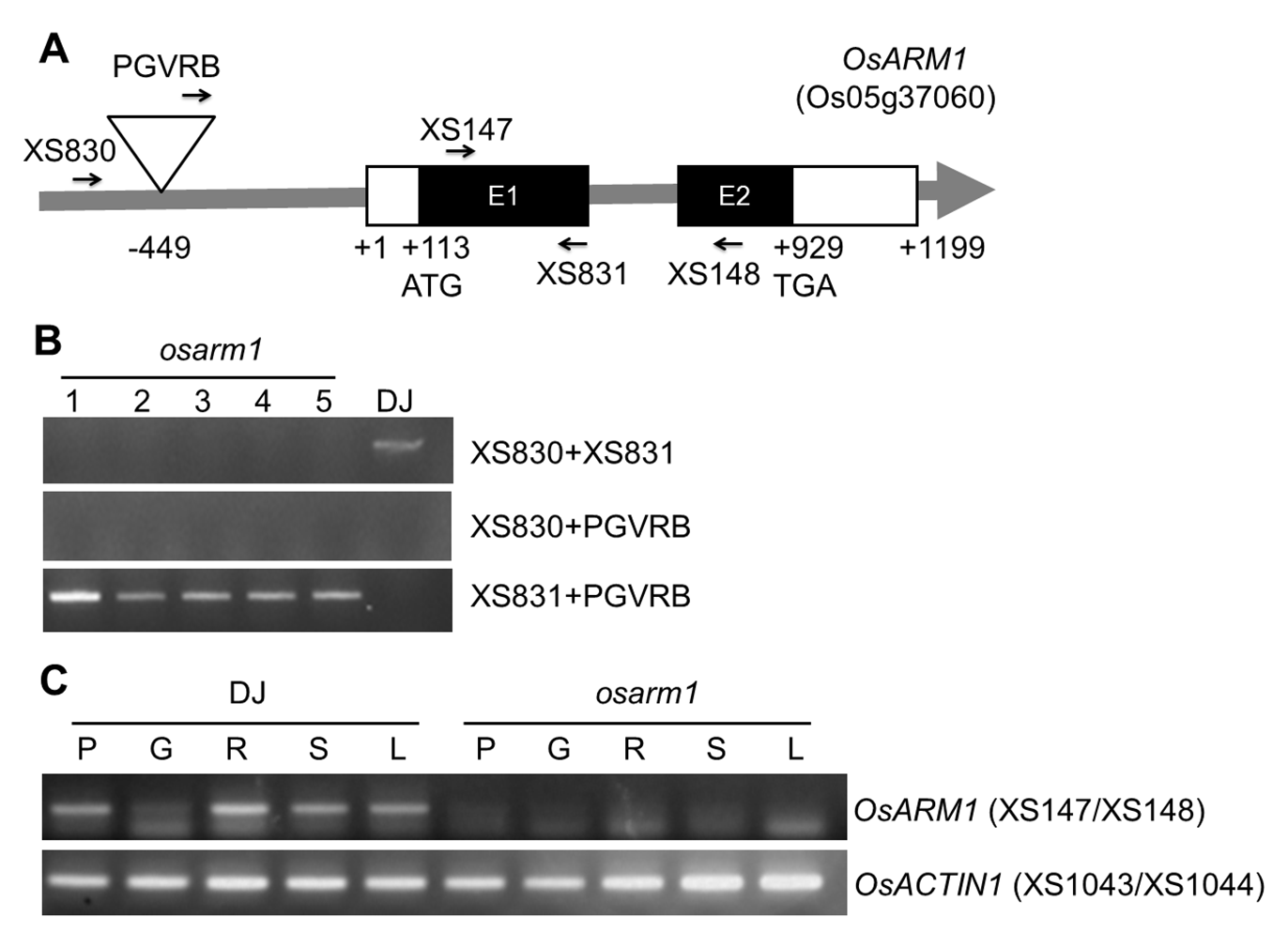

Supplement: Figure S2 — Identification of the osarm1 T-DNA insertion mutant. (A) Location of T-DNA insertion site in OsARM1 in the osarm1 mutant (Os05g37060). Primer pairs XS830/XS831, XS830/PGVRB, and XS831/PGVRB were used to genotype the mutant. Primer pair XS147/XS148 was used to determine OsARM1 expression levels. E1 and E2 denote exon 1 and exon 2, respectively. (B) Genotyping analysis of osarm1 mutant by tri-primer PCR. All five independent plants are homozygous osarm1 mutants. (C) RT-PCR showing the expression levels of OsARM1 in various organs of wild type (DJ) and osarm1. OsACTIN1 was used as a reference gene. P, panicle; G, grain; R, root; S, stem; L, leaf blade. [file Image2.TIF]

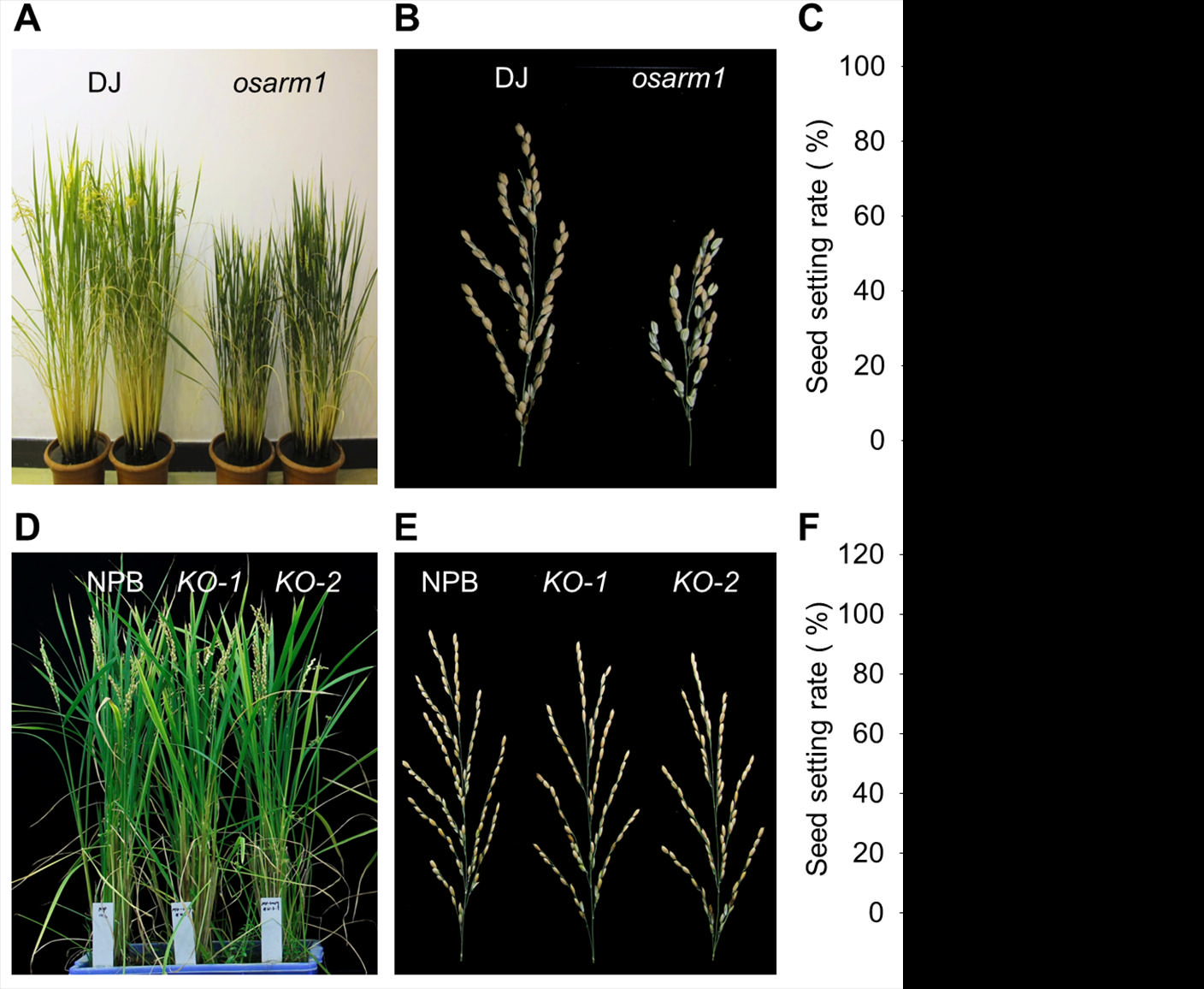

Supplement: Figure S3 — Phenotypes of OsARM1 mutants. The osarm1 seedlings showed reduced plant height (A), panicle number (B), and seed setting rate (C) compared to the wild-type DJ. But all the phenotypes mentioned above of OsARM1-KO lines were the same as wild-type NPB (D–F). Asterisks indicate significant differences from wild type (**P < 0.01 by Student's t-test). [file Image3.TIF]

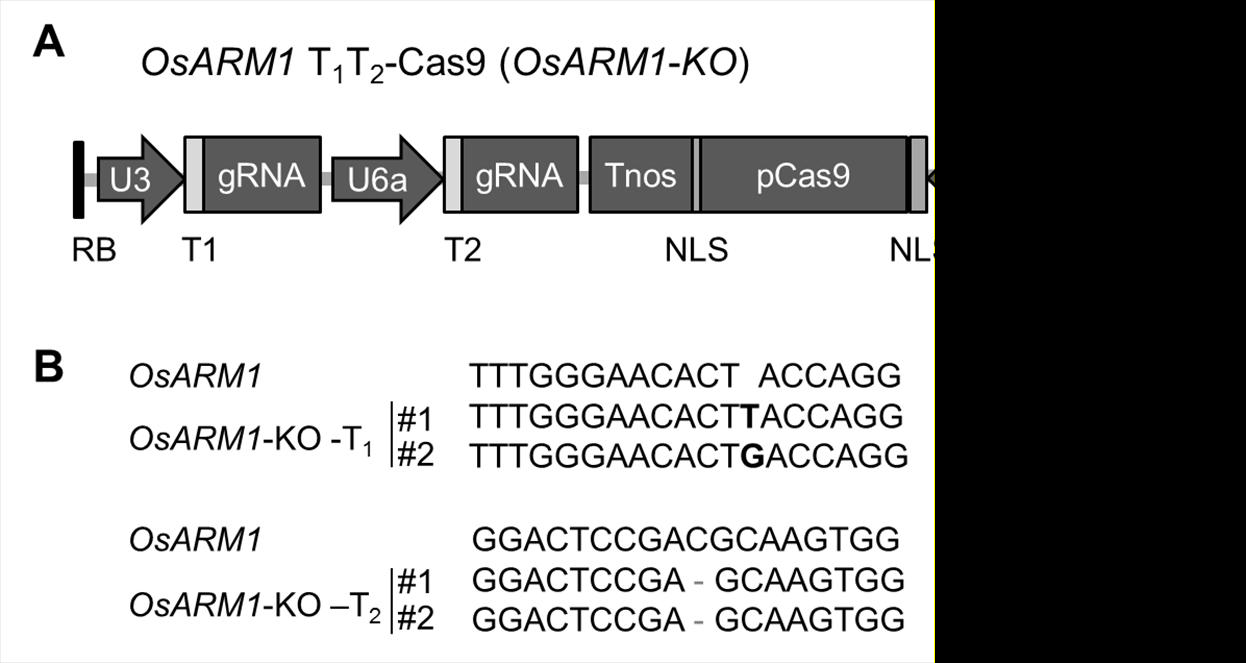

Supplement: Figure S4 — Generation of OsARM1-KO transgenic lines. (A) Schematic diagram of the vector OsARM1 T1T2-Cas9 (OsARM1-KO). (B) Genome editing efficiency of OsARM1-KO transgenic rice lines. Two independent transgenic lines containing a 1-bp insertion and 1-bp deletion, respectively, at the expected cleavage site were obtained. [file Image4.TIF]

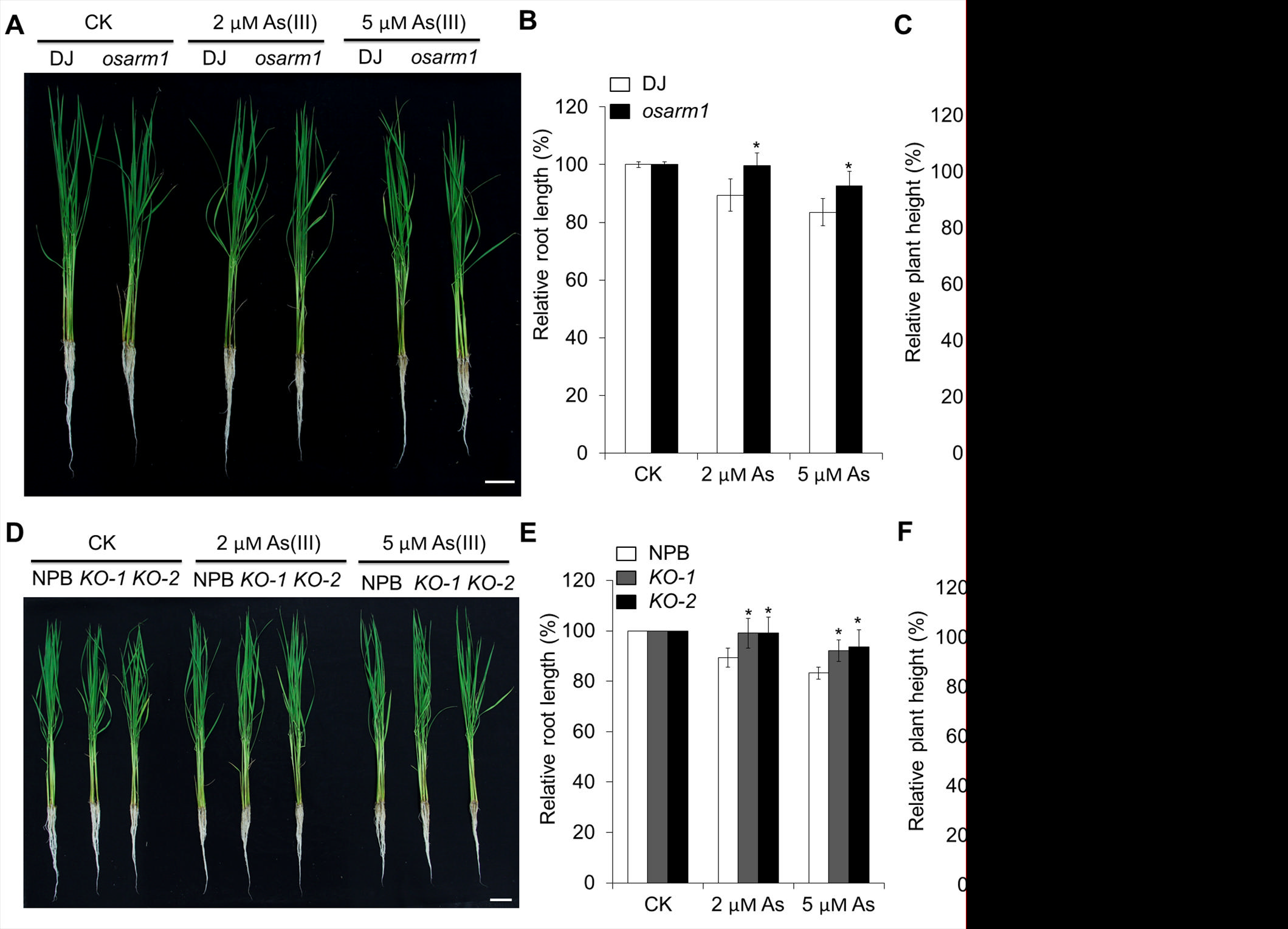

Supplement: Figure S5 — Knockout of OsARM1 confers enhanced tolerance to low concentrations of As(III). (A,D) Phenotypes of 2-week-old wild-type DJ and osarm1 or wild-type NPB and OsARM1-KO transgenic lines (KO-1 and KO-2) plants grown on Kimura B nutrient solution without (CK) or with 2 or 5 μM As(III) for 14 days. During the treatment, renewed the treatment solution on the 7th day. (B,C) Relative root elongation (B) and relative plant height (C) of DJ and osarm1 plants after As(III) treatment in (A). (E,F) Relative root elongation (E) and plant height (F) of NPB and OsARM1-KOs (KO-1 and KO-2) after As(III) treatment in (D). Asterisks indicate significant differences from wild type (*P < 0.05; **P < 0.01 by Student's t-test). Bars = 4 cm. [file Image5.TIF]

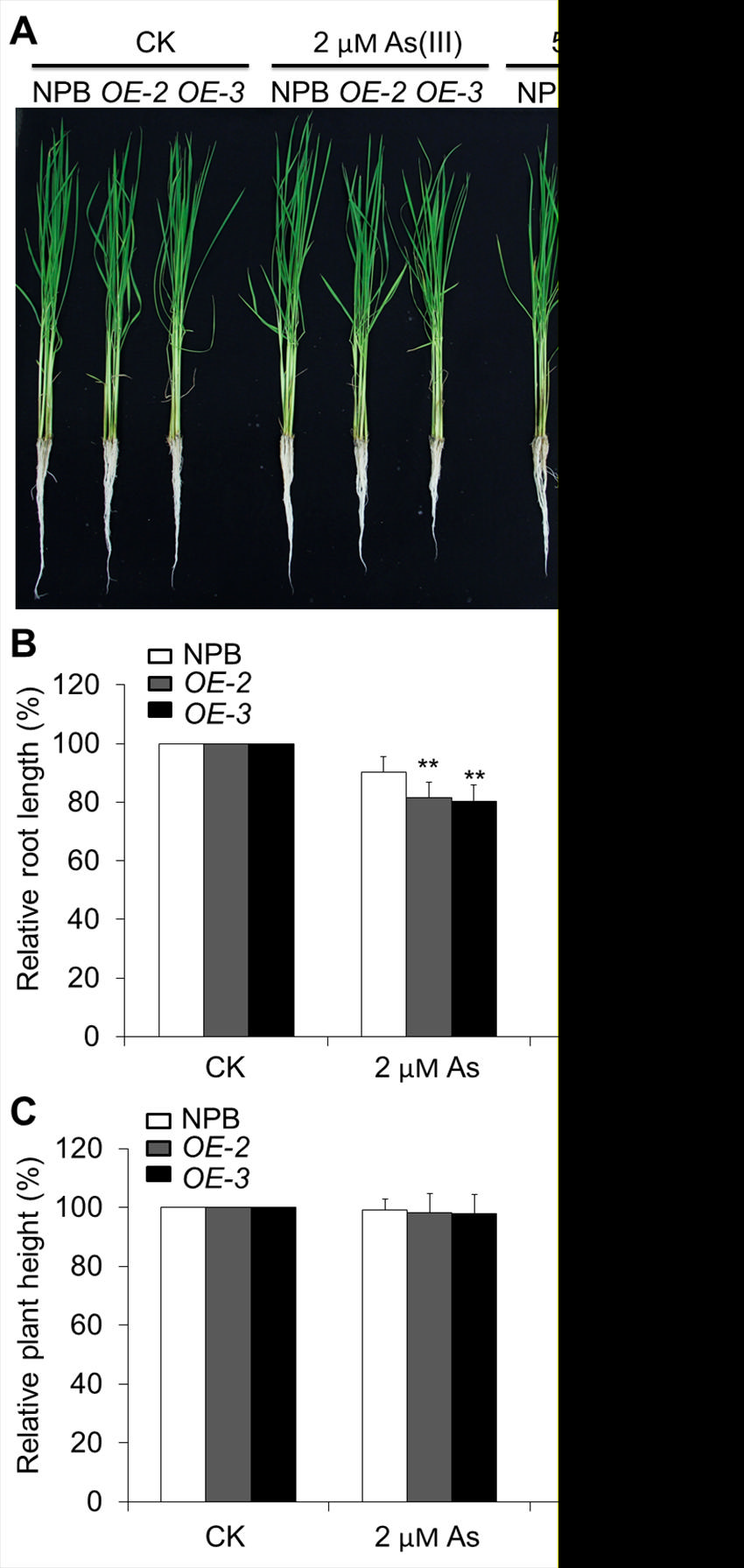

Supplement: Figure S6 — Overexpression of OsARM1 attenuates tolerance to low concentrations of As(III). (A) Images of wild-type NPB and OsARM1-OE transgenic lines (OE-2 and OE-3) treated without (CK) or with As(III). Two-week-old seedlings were grown on Kimura B medium or Kimura B medium containing 2 or 5 μM As(III) for 14 days. During the treatment, renewed the treatment solution on the 7th day. Bars = 4 cm. (B,C) Relative root elongation (B) and relative plant height (C) of NPB and OsARM1-OE transgenic lines (OE-2 and OE-3) after As(III) treatment. Asterisks indicate significant differences from wild type (*P < 0.05; **P < 0.01 by Student's t-test). [file Image6.TIF]

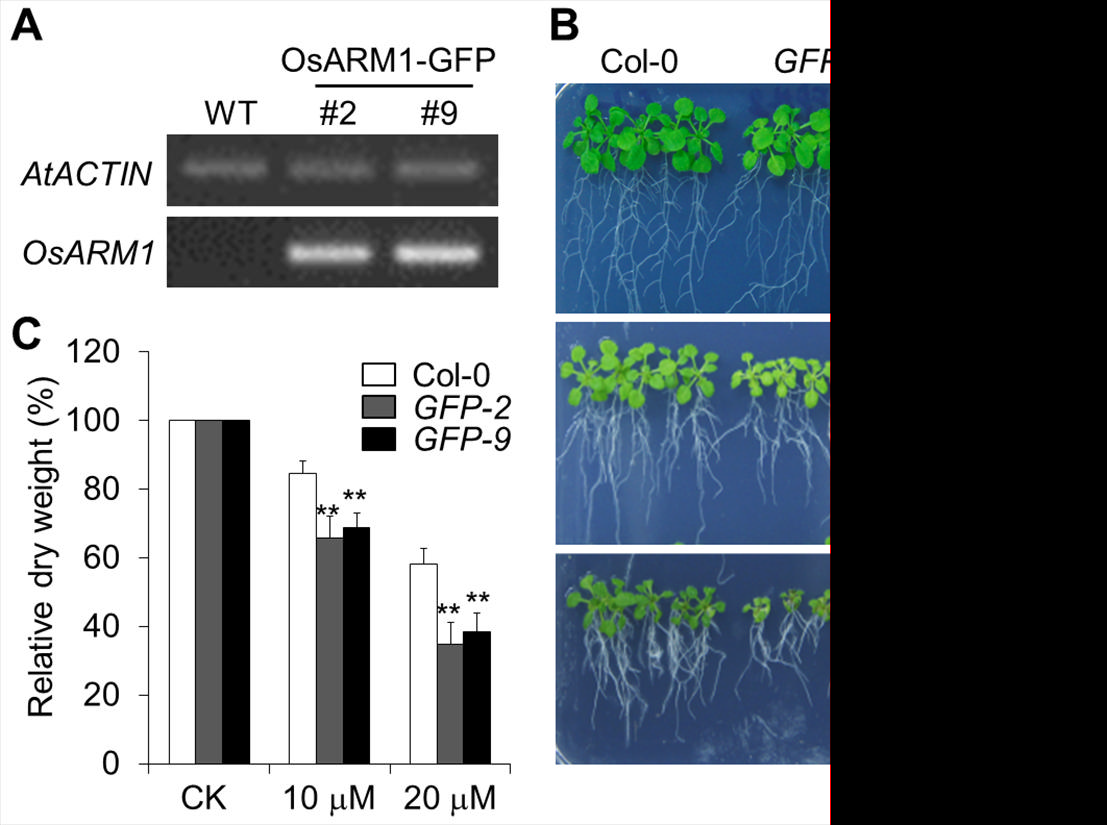

Supplement: Figure S7 — Phenotypic analysis of transgenic Arabidopsis expressing OsARM1-GFP in response to As stress. (A) RT-PCR detected the expression level of OsARM1 in 35S::OsARM1-GFP transgenic Arabidopsis. (B) Wild type (Col-0) and OsARM1-GFP transgenic lines (GFP-2 and GFP-9) were germinated on 1/2 MS medium for 7 days. The seedlings were subsequently transferred to 1/2 MS medium (CK) or 1/2 MS medium containing 10 or 20 μM As(III) and grown vertically. The images were taken at 2 weeks after treatment, and the relative dry weights were calculated thereafter (C). The experiments were repeated three times (biological replicates), and >10 plants were used for each genotype in a single experiment. Data are means ± SD (n = 3) of three biological replicates. Asterisks indicate significant differences from wild type (**P < 0.01 by Student's t-test). [file Image7.TIF]

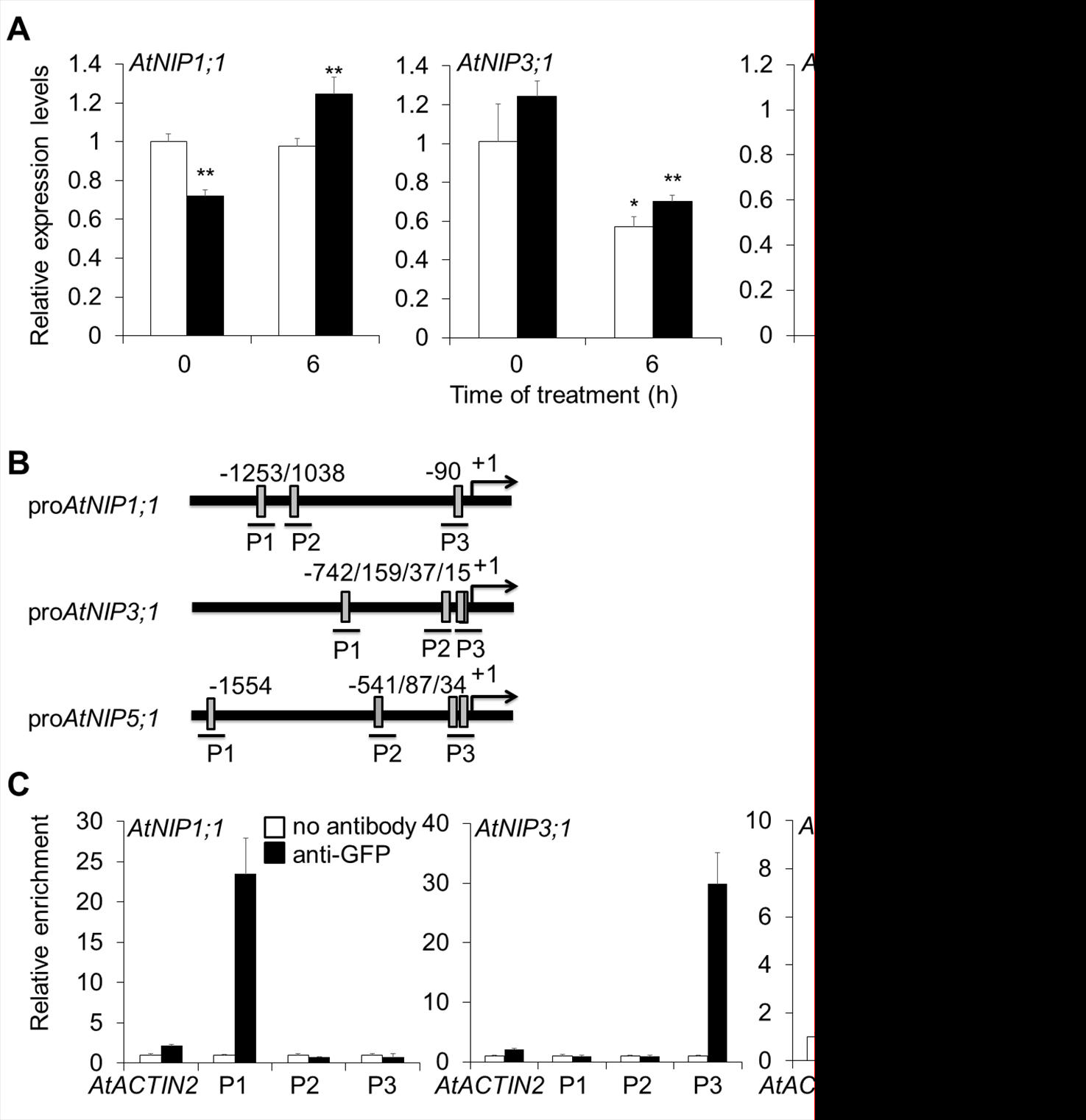

Supplement: Figure S8 — OsARM1 directly interacts with the promoters of AtNIP1;1, AtNIP3;1, and AtNIP5;1 in Arabidopsis. (A) Expression of As-related transporter genes in Arabidopsis. Total RNA was extracted from the wild-type Col-0 and GFP-9 plants grown on half-strength MS medium for 14 d then transferred to filter paper soaked with 40 μM As(III) for 0 and 6 h. The expression levels of AtNIP1;1, AtNIP3;1, and AtNIP5;1 were examined by qRT-PCR analyses. AtACTIN2 was used as a reference gene. Asterisks indicate significant differences from wild type (*P < 0.05; **P < 0.01 by Student's t-test). (B) Schematic diagram of the potential AC-I element (ACC(A/T)A(A/C)) in the promoter sequences of AtNIP1;1, AtNIP3;1, and AtNIP5;1. Lines under the boxes indicate sequences detected by ChIP-qPCR. Numbers indicate the nucleotide positions relative to the corresponding translational start site (ATG), which is shown as +1. (C) ChIP-qPCR analyses showing the in vivo interaction between OsARM1 and the predicted AC-I element in the promoters of AtNIP1;1, AtNIP3;1, and AtNIP5;1. Protein/DNA complexes isolated from the whole seedlings of OsARM1-GFP transgenic Arabidopsis were immunoprecipitated with or without the anti-GFP antibody. For each promoter, various DNA fragments were used to determine the enrichment of the DNA fragment containing the AC-I element. A promoter fragment of AtACTIN2 was used as a negative control. The experiment was repeated three times with similar results. [file Image8.TIF]

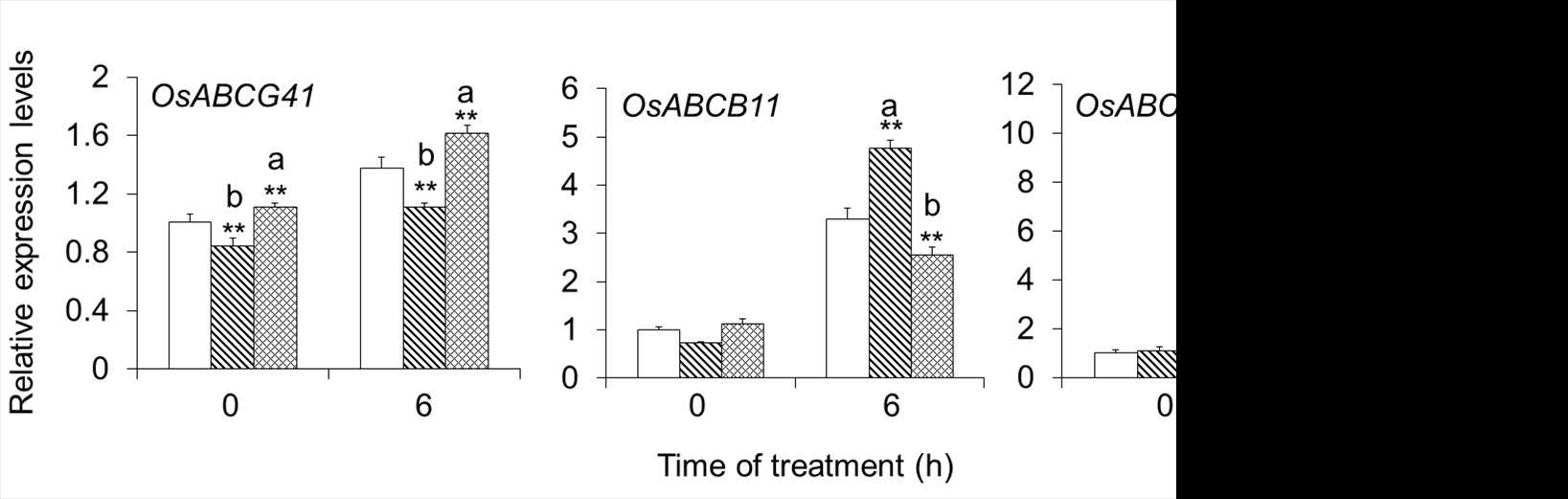

Supplement: Figure S9 — Expression of As-responsive transporter genes in wild type, OsARM1-KO, and OsARM1-OE. Total RNA was extracted from the shoots of 2-week-old seedlings (wild-type NPB, OE-2, and KO-1) treated with Kimura B nutrient solution containing 50 μM As(III) for 0 and 6 h. The expression levels of OsABCG41, OsABCB11, and OsABCC14 were examined by qRT-PCR analyses. OsGAPDH was used as a reference gene. The experiment was repeated three times with similar results. Asterisks indicate significant differences from wild type (*P < 0.05; **P < 0.01 by Student's t-test). “a” and “b” indicate values that are significantly higher or lower, respectively, in OE-2 or KO-1 than in wild type. [file Image9.TIF]

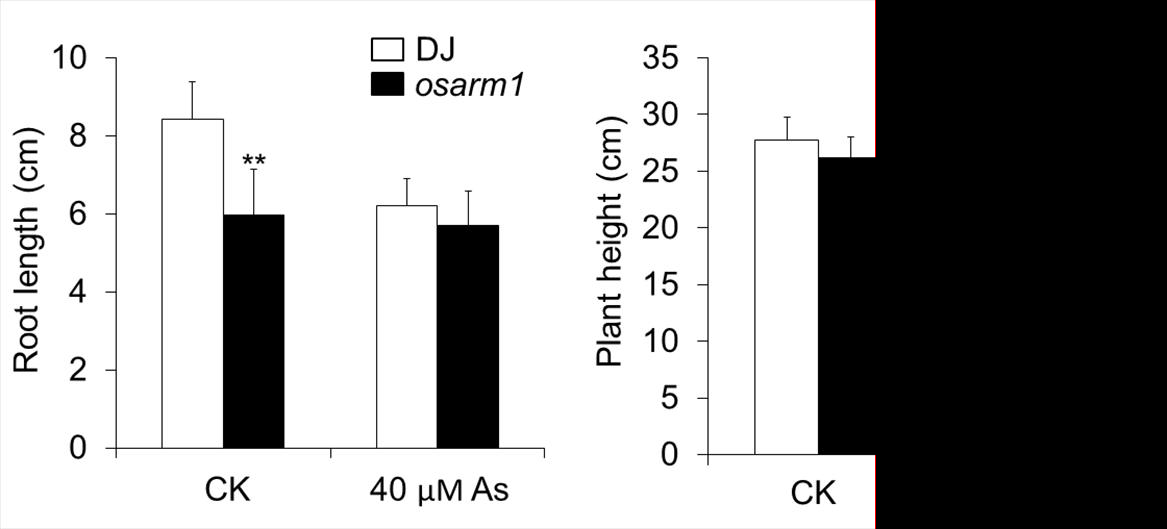

Supplement: Figure S10 — Absolute values of root length (A) and plant height (B) of DJ and osarm1 in Figure 4A. Two-week-old wild-type DJ and osarm1 plants grown on Kimura B nutrient solution without (CK) or with 40 μM As(III) for 7 days. The experiment was repeated three times with similar results. Asterisks indicate significant differences from wild type DJ (*P < 0.05; **P < 0.01 by Student's t-test). [file Image10.TIF]
